# The association between orthorexia nervosa and social media use: A systematic review and meta-analysis

**DOI:** 10.1371/journal.pmen.0000758

**Published:** 2026-10-21

**Authors:** Anna-Marina Nakhl, Muhammad Ahmad Barakat, Jana Kotaich, Elssy Elleik, Ahmad Afyouni, Linda Abou Abbas

**Affiliations:** 1 MEDICA Research Investigation, Hadath, Lebanon; 2 Faculty of Medical Sciences, Lebanese University, Hadath, Lebanon; 3 Faculty of Medical Sciences, Caucasus International University, Tbilisi, Georgia; 4 INSPECT-LB (Institut National de Santé Publique Epidémiologie Clinique et Toxicologie-Liban), Beirut, Lebanon; 5 Neuroscience Research Center, Faculty of Medical Sciences, Lebanese University, Beirut, Lebanon; BronxCare Health System, UNITED STATES OF AMERICA

## Abstract

Orthorexia nervosa (ON) is a newly described mental disorder characterized by an extreme obsession with the consumption of healthy foods, resulting in anxiety and impaired functionality. This systematic review and meta-analysis aims to synthesize the association between social media use and orthorexic tendencies among adolescents and adults. PubMed, Web of Science, Embase, Scopus, and PsycINFO were searched through January 2026. Original studies assessing the association between social media use and ON were included. Zero-order correlations between social media and orthorexia measures were pooled using Fisher’s z under a random-effects (REML) model; heterogeneity, subgroup and meta-regression, sensitivity and publication-bias analyses were performed; certainty was rated with GRADE. Of 33 included studies, 22 contributed to meta-analysis of the correlation between social media use and ON symptoms, yielding a small positive pooled correlation (r = 0.20, 95% CI 0.15–0.25; I² = 84.3%; very low certainty by GRADE). This association remained stable across leave-one-out, subgroup, and sensitivity analyses (r = 0.18–0.20), and after adjustment for potential publication bias (bias-adjusted r = 0.17). A secondary analysis of two studies with reconstructable 2 × 2 data suggested greater odds of ON among heavier users (OR = 1.57, 95% CI 1.25–1.97). Pooled ON prevalence differed sharply with the diagnostic threshold (39.9% at the ORTO-15 cutoff below 35 versus 65.5% below 40), reflecting extreme heterogeneity (I² > 90%) across studies. Narrative synthesis indicated that the type of content consumed was more consistently associated with ON than platform or duration of use alone Social media use is associated with greater orthorexic tendencies. However, because all included studies were cross-sectional and overall certainty of evidence was very low, causality and directionality cannot be established and reverse causality remains plausible. Longitudinal and experimental research is needed to clarify the direction of this relationship.

## Introduction

Orthorexia is defined by an obsessive and extreme fixation on consuming exclusively “healthy” and “pure” foods with an interest in the cleanliness and quality of the diet [1]. The term “Orthorexia”, Greek for “right appetite”, was first brought to the spotlight in 1997 by Dr. Steven Bratman, a holistic medical practitioner, in Yoga Journal, who noticed orthorexic tendencies or excessive healthy eating among his patients [2–5]. However, Orthorexia Nervosa (ON) emerged afterwards to describe an added factor of severity and rigidity that distinguishes pathological orthorexic behaviors from non-pathological interest in healthy eating, with it being proposed as a distinct condition in the 1990s ^6^. It is clinically significant in that this obsession is now amplified with heightened anxiety, distress, and daily dysfunction due to pathologic dietary restrictions distinguishing it from healthy interest in nutritious eating [6,7]. This heightened anxiety is related to the patient’s food choices, where distress occurs when deviations from the strictly laid out dietary rules are made, leading to multiple severe nutritional deficiencies as well as social withdrawal and impaired functioning, leading to decreased quality of life [4].

It should be noted that ON is not documented or represented in either the Diagnostic and Statistical Manual of Mental Disorders (DSM-V-TR) or the International Classification of Diseases (ICD-11) and thus remains a proposed rather than officially recognized psychiatric condition [4]. Therefore, there are no officially standardized criteria for the diagnosis of ON, but researchers have proposed set of criteria usually includes features such as high preoccupation with the purity and quality of food, intense focus on the nutritional value and content of the meal, and extremely rigid dietary rules and restrictions, all of which culminate in a significant effect on daily life and functionality [8]. These criteria can be supplemented with heightened anxiety and distress due to deviating from the rigid diet, leading to psychological impairment and disruption in educational, occupational, and social activities [9]. Some researchers proposed that ON might differ from other eating disorders in terms of reduced weight gain or body image distortion. However, the specific role of body image in differentiating ON from other disordered eating practices remains an area of active investigation, a topic that will be discussed in this systematic review [6].

Some of the challenges in the identification of ON (other than lack of documentation in DSM-V and ICD-11 and lack of standardized criteria) include blurred boundaries with other eating disorders and similar mental health conditions, symptom overlap, and reliance on self-report mechanisms using the ORTO-15 scales or similar instruments, which have demonstrated psychometric limitations [6]. These challenges in identification & assessment are of utmost importance, as individuals exhibiting severe orthorexic behaviors may experience complications including potential malnutrition, digestive issues, mood disorders, and intense social isolation, though definitive longitudinal studies establishing direct causal pathways in general populations are still needed.

In 2023, 47 eating disorder researchers and multidisciplinary treatment specialists came together to set a sort of consensus document for proposed diagnostic criteria for ON with an important point being that the previously mentioned criteria have to persist for around 6 months unless there is severe impairment of health (such as severe malnutrition), in which case, they should persist for 3 months [5]. This consensus document also proposes exclusion criteria for ON assessment, including being on clinical dietary prescriptions, the presence of clinical conditions that motivate food selection and exclusion, and whether food exclusion is due to economic conditions, values, cultural, religious beliefs, or delirious ideas [5].

Understanding of ON has evolved to encompass how specific health-related societal pressures are associated with disordered eating patterns, which include social media content and problematic social media use. Social media emerged in 2004 and has evolved into a dominant platform for communication and information sharing [10,11].

Time spent on social media platforms is rapidly increasing and this high usage is highly linked to multiple negative mental health consequences such as suicidality, loneliness, anxiety, body image issues, and a notably high incidence of disordered eating behaviors (correlated with high usage rates [11,12].

Understanding the characteristics and consequences of problematic social media use is particularly relevant to ON research, as engaging excessively with health-related and diet-focused content on social media platforms may be associated with developing orthorexic tendencies. The overlapping features of compulsive behavior, anxiety and body dissatisfaction in both ON and problematic social media use suggest potential shared mechanisms worth investigating. People who negatively use social media, as in, have a sort of addiction, are therefore described as having “problematic social media use”, an excessive use characterized by negative impacts on daily functioning and emotional distress when not engaging with these platforms [13]. Problematic social media users are more likely to be anxious, depressed, socially isolated, have poorer sleep quality, have lower self-esteem, and higher body image dissatisfaction [14]. Users who engage with health-related content having significant correlations to eating disorders and body dissatisfaction [10,11].

Among different types of social media platforms, Instagram use has been associated with higher rates of orthorexic tendencies, potentially related to its visual focus, usage patterns, and the layout of posts and content on the platform [14,15]. Food-related posts on Instagram showcasing balanced meals without processed sugars or added fats are pushed further by the algorithm due to seeing acceptance and approval compared to unhealthy food posts [16]. Exposure to such content may contribute to difficulty for some adolescents and young adults in discerning between disordered and healthy eating. Research has found associations between Instagram use and orthorexic tendencies, particularly among individuals who show great interest in their appearance and diet, though causal relationships have not been definitively established [17]. Therefore, social media, specifically Instagram use has been among the platform exposures most consistently associated with orthorexic tendencies [18].

According to some studies, 49% of individuals who follow health food accounts on Instagram score above the threshold on screening instruments for orthorexic tendencies, though it is important to note that such screening tools may over-pathologize healthy interest in nutrition [17]. Social media by its nature creates trends and popularizes accounts that follow such trends. Most of these trends promote unrealistic standards for overly healthy and disordered eating, leading to adolescents falling into this trap as they aren’t able to discern due to a lack of digital literacy, which contributes to the development of orthorexic behaviors. Research suggests associations between exposure to such trends and the development of orthorexic behaviors, through the mechanisms and causal pathways, require further investigation [19–21].

Two essential theories explain the relationship between media exposure, body image and eating behavior, providing a theoretical foundation for understanding the association between social media use and ON. First, the sociocultural theory [22] offers a widely accepted framework, potentially explaining the complex relationship between media, body image and dissatisfaction, and eating disorders. According to this theory, one’s close interaction with individuals influences their perceptions [23]. Furthermore, this can sometimes result in the adoption of unhealthy and even extreme disordered eating practices in the aim of pursuing thinness [24,25]. These effects don’t appear abruptly but rather develop with time following repeated exposure to unrealistic beauty standards in individuals rather vulnerable to these exposures [24].

Second, the objectification theory [25], developed by Fredrickson and Robertson in 1997, points out that women are often presented in the media in a way that focuses solely on their appearance, often in a sexualized way. Under this framework, repeated exposure is proposed to lead to the internalization of this external view and to “self-objectification”, a process where one’s physical appearance dominates their self-assessment. This constant body surveillance increases body shame and anxiety, which makes the individual more vulnerable to body dissatisfaction as well as disordered eating behaviors, such as restrictive eating [26,27].

These theoretical frameworks help explain the increased internalization of thin ideals, appearance comparison and body dissatisfaction among internet and social media users [27,28], while also explaining the mediating effect of body dissatisfaction between internalization and ON [29–31]. Prospective and longitudinal studies have found body dissatisfaction to be one of the most stable and reliable risk factors for developing eating disorders [32–35].

Across this literature the type of content a user is exposed to is more consistently associated with orthorexic tendencies than the time spent on the platform [36].

The interplay between social media use (and addiction) and ON is highly important to be understood in order to develop targeted interventions, raise awareness, and potentially reduce the prevalence of orthorexic tendencies among at-risk groups [11,18]. Most recently, Awad and colleagues conducted a mixed-methods scoping review examining the relationship between social media and ON, synthesizing 31 studies published between 2017 and 2024 identified through a search conducted in August 2023 and updated in September 2024 [37]. Following PRISMA-ScR guidance and the Mixed Methods Appraisal Tool, that review incorporated quantitative, qualitative, and mixed-methods evidence and concluded, based on the heterogeneity of populations, methods, and outcome measures across studies, that a meta-analysis was not supportable and that findings should instead be synthesized narratively. While this scoping review offers a valuable descriptive map of the literature, several gaps remain. First, its search window closed in September 2024, before a substantial number of newer studies—including several published in 2025—became available. Second, its scope-driven inclusion of qualitative and mixed-methods designs, while appropriate for a scoping review, means that quantitative associations were not appraised using a formal risk-of-bias tool specific to observational designs, nor pooled statistically. The present review addresses these gaps by restricting inclusion to quantitative studies employing validated ON measures, applying the Newcastle-Ottawa Scale to formally appraise methodological quality, and conducting a random-effects meta-analysis of correlational effect sizes where sufficient homogeneity across studies allowed pooling. In doing so, this review extends the descriptive synthesis provided by Awad et al. [37] with a quantitative estimate of the pooled association and an updated evidence base.Therefore, in this systematic review, we aim to answer the following question: “What is the relationship between social media use and orthorexic behaviors, and does social media use contribute to the prevalence and severity of orthorexic tendencies?”

## Methods

### Study design and protocol registration

A review proposal was registered with the International Prospective Register of Systematic Reviews (PROSPERO, registration number CRD42025643104). This study was designed, conducted, and reported according to the Preferred Reporting Items for Systematic Reviews and Meta‐Analyses (PRISMA) Statement [38]. There was no need for Ethics Committee approval, as this is a systematic review.

### Eligibility criteria

The eligibility criteria were structured using the Population, Exposure, Comparator, and Outcome (PECO) framework:

- **Population (P):** Adolescents and adults who use social media, regardless of gender, occupation, or geographic location.
- **Intervention/Exposure (I):** Social media use (e.g., Instagram, Facebook, TikTok, X, and other platforms).
- **Comparator (C):** No uniform comparator was required because the included studies were predominantly observational and differed substantially in how social media engagement was assessed. The comparator was therefore defined within each individual study as the contrast between participants with relatively higher versus lower levels of social media engagement, according to that study’s specific operationalization. Depending on the study, social media engagement was assessed using measures such as duration or frequency of use, scores reflecting problematic or addictive social media use, type of platform used, or type of content consumed (e.g., health- or nutrition-related versus non-health-related content). Thus, the comparator was not assumed to be equivalent across studies. **Outcome (O):** Prevalence and severity of ON symptoms, measured through validated tools.

Studies were eligible if they met all the following criteria:

- included adolescent or adult social media users;
- examined social media use as the primary exposure;
- assessed orthorexia nervosa using a validated measurement instrument; and
- cross-sectional, cohort, case-control, and experimental designs reporting data on the association between social media use and ON

Studies were excluded if they had any of the following:

- focused exclusively on children;
- did not assess social media use;
- did not evaluate orthorexia nervosa;
- used non-validated orthorexia assessment tools;
- were reviews, editorials, commentaries, conference abstracts without full-text publications, protocols, or other non-original research;

### Information source and search strategy

A comprehensive electronic search was conducted in PubMed/MEDLINE, Embase, Scopus, Web of Science, and PsycINFO in January 2025 and updated on January 2026. The search strategy combined controlled vocabulary (e.g., Medical Subject Headings [MeSH]) and free-text terms related to orthorexia nervosa, orthorexia, obsessive healthy eating, and social media, with database-specific adaptations. Boolean operators (“AND” and “OR”) were used to combine search terms. Grey literature (e.g., dissertations, institutional reports, and theses) was also searched; however, only studies meeting the same eligibility and methodological quality criteria applied to peer-reviewed publications were considered for inclusion. Citation tracking was not performed. The complete search strategies for all databases are provided in S1 Text.

Study selection Records identified through the database searches were imported into Rayyan for duplicate removal and screening. Two reviewers (AN and MB) independently screened titles and abstracts, followed by independent full-text assessment by MB and EE (Full text screening available in S1 Data). Disagreements regarding study eligibility were resolved through discussion and, when necessary, consultation with a third reviewer.

No automated screening or artificial intelligence-assisted selection tools were used beyond Rayyan’s duplicate detection. When eligibility or relevant quantitative data could not be determined from the published report, corresponding authors were contacted for clarification.

### Data extraction

Data extraction was performed independently by the reviewers using a standardized extraction form that was piloted before full implementation to ensure consistency.

The following information was extracted:

- study characteristics (authors, publication year, country, and study design);
- participant characteristics (sample size, age, body mass index, sex distribution, and recruitment setting);
- exposure characteristics (social media platform, assessment instrument, frequency or duration of use, and content type);
- orthorexia assessment characteristics (instrument used, scoring method, prevalence, symptom severity, and diagnostic thresholds); and
- study findings relevant to the association between social media use and orthorexia nervosa.

For quantitative synthesis, studies were eligible if they reported a zero-order Pearson or Spearman correlation between a continuous measure of social media use and a continuous orthorexia score, together with the corresponding sample size or sufficient information to derive the effect estimate. When multiple eligible effect sizes were reported from the same study, a predefined decision rule was applied to retain a single independent estimate for the primary meta-analysis, while alternative estimates were summarized descriptively. When required information was unavailable, corresponding authors were contacted.

### Methodological quality assessment

#### Appraisal of orthorexia measurement.

Because no orthorexia instrument is formally validated against a diagnostic standard, measurement properties were treated as a source of bias in their own right rather than as a background caveat. For every included study, we recorded the instrument used, its scoring direction, the cutoff applied, and the internal consistency reported in that sample. Two measurement characteristics were pre-specified as moderators of the pooled correlation: the instrument family (ORTO-11, ORTO-15 and ORTO-R versus DOS, TOS, ONI and EHQ) and the scoring direction (reverse-scored versus normally scored). Where an instrument was applied at more than one cutoff across studies, prevalence was reported separately by cutoff and was not pooled across thresholds. This allowed the influence of measurement choice on both the pooled association and the estimated prevalence to be quantified rather than assumed.

The Newcastle-Ottawa Scale adapted for cross-sectional studies was used to assess the risk of bias [39]. This scale uses a star system with a maximum of ten stars to evaluate a study in three domains: selection of participants, comparability of study groups and ascertainment of outcomes of interest [40]. Risk of bias was independently assessed by two reviewers, with disagreements resolved by consensus or adjudication by a third reviewer. Studies were categorized as having low (7–10 stars), moderate (4–6 stars), or high (0–3 stars) risk of bias according to predefined score thresholds.

#### Statistical synthesis.

Each included study was assigned a priori to one or more synthesis tracks according to the data it reported. A study entered the correlational meta-analysis if it reported a zero-order Pearson or Spearman correlation between a continuous social media measure and a continuous orthorexia score; the dichotomous analysis if a 2 × 2 table of orthorexia caseness by level of social media use was reported or could be reconstructed from published band prevalences; a prevalence stratum if it reported orthorexia caseness against a stated instrument cutoff; and the narrative synthesis if none of these applied. A study could contribute to more than one track, which is why the summed track counts exceed the number of included studies.

Pearson correlation coefficients (r) were transformed to Fisher’s z values prior to pooling. Random-effects meta-analysis using the restricted maximum likelihood (REML) estimator was performed to account for anticipated between-study heterogeneity. Correlations derived from reverse-scored ORTO-11 or ORTO-15 instruments were multiplied by −1 before analysis so that positive effect estimates consistently indicated greater social media use associated with greater orthorexia symptomatology. Pooled estimates were subsequently back-transformed to Pearson’s r for interpretation.

Between-study heterogeneity was evaluated using Cochran’s Q, τ², and I² statistics. Prespecified subgroup analyses and mixed-effects meta-regression were conducted to investigate potential sources of heterogeneity according to orthorexia assessment instrument, scoring direction, social media construct, correlation type, publication year, and sample size.

Robustness was examined by leave-one-out analysis, influence diagnostics (externally studentized residuals, DFFITS, Cook’s distance, covariance ratio, hat values and leave-one-out heterogeneity) and a Baujat plot. Eight pre-specified exclusion models were fitted, each omitting in turn Spearman-derived coefficients, composite measures, the single very small sample, studies whose effect sign had to be inferred, content-specific measures, the pregnancy sample, studies rated at high risk of bias on the Newcastle-Ottawa Scale, and all measures other than social-media-addiction scales. Small-sample adjusted (Knapp-Hartung) standard errors were used for subgroup and meta-regression models. Small-study effects were assessed by contour-enhanced funnel plot, by meta-regression of effect size on log sample size, and by the Duval-Tweedie trim-and-fill procedure. Certainty of evidence for the primary pooled correlation was rated with GRADE across the domains of risk of bias, inconsistency, indirectness, imprecision and publication bias.. Where available, odds ratios comparing orthorexia prevalence across different levels of social media use were synthesized separately using random-effects meta-analysis. Prevalence was pooled only within strata sharing both the same instrument and the same diagnostic cutoff, using a random-effects model on logit-transformed proportions. Estimates were never combined across instruments or across cutoffs, and where an instrument was applied at more than one cutoff across studies its estimates are reported as a range rather than pooled. Given the extreme within-stratum heterogeneity observed, all pooled prevalence figures are presented as descriptive.

All statistical analyses were performed using RStudio version 2026.05.1 + 225 using the metafor package.

## Results

### Search result and study selection

A total of 880 articles were retrieved from five different databases. After removing duplicates and screening titles and abstracts, 43 articles were identified. Following full-text screening, 33 articles met the eligibility criteria and were included (Fig 1). The studies included were predominantly published within the past five to seven years. This reflects the increasing scholarly focus on ON during this recent period, rather than an intentional exclusion of earlier work.

**Fig 1:**
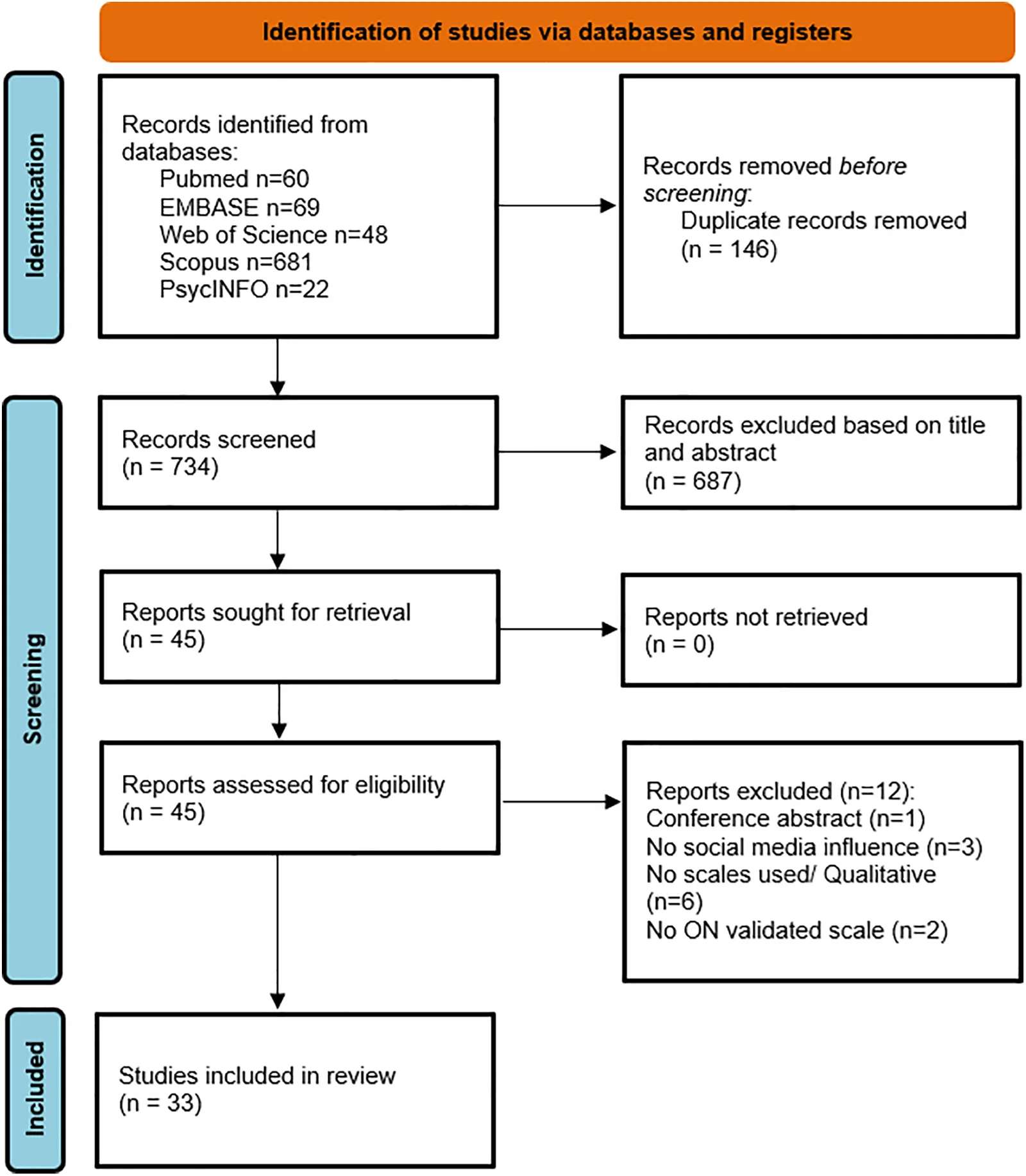
PRISMA flowchart illustrating the study selection process.

The Newcastle-Ottawa scale adapted for cross-sectional studies was used to assess the quality of each study. Total scores ranged from 3 to 7 stars. Among the 33 included studies, one was at high risk of bias (Daniel & Gendle 2025, 3 stars) [41], twenty one were at moderate risk (4–6 stars) [14,29,30,42–58] and eleven were at low risk (7–10 stars) [31,59–68]; individual domain and total scores for every included study are reported in S1 Table.

All 33 articles had a cross-sectional study design, with two articles incorporating other study designs such as an experimental [31] and a descriptive [53] study design. Among the identified studies, most were conducted in Turkey. The remaining studies were conducted in different countries across the world (Table 1).

**Table 1:** Characteristics of Studies Examining the Relationship Between Orthorexia Nervosa and Social Media Usage, including Country, Population, Mean Age, Mean BMI and Gender.

Author (Year)	Title	Country	N	Population	Mean Age + SD	Mean BMI + SD	Gender (Male %)
Dobos (2024)	Mindful eating and orthorexia nervosa: How do they interact?	Hungary	551	University students recruited via Facebook student groups	22.6 ± 5.6	22.74 ± 5.61	127 males (23.1%)
Awad (2024)	Association of Social Media Use Disorder and Orthorexia Nervosa Among Lebanese University Students: The Indirect Effect of Loneliness and Factor Structure of the Social Media Use Disorder Short Form and the Jong-Gierveld Loneliness Scales	Lebanon	363	University students	22.65 ± 3.48	not asked	139 (38.3%)
Staskiewicz-Bartecka (2024)	The Impact of Social Media and Socio-Cultural Attitudes toward Body Image on the Risk of Orthorexia among Female Football Players of Different Nationalities	Poland, Turkey, India	142	Female football players from sports clubs in Poland, Turkey, and India	20.7 ± 3.79	21.4 ± 2.50	0 (0%)
Kalender (2024)	A Study on Health Nutrition Obsession, Social-Physique Anxiety, and Social Media Addiction of Young Individuals	Turkey	400	Individuals aged 18–29 in Isparta, Turkey	74% in the 18–24 age group	64% in the 19-24.9 BMI range	123 (30.7%)
Piko (2024)	Orthorexic Tendency in Light of Eating Disorder Attitudes, Social Media Addiction and Regular Sporting Among Young Hungarian Women	Hungary	310	Hungarian young women who were especially interested in a healthy lifestyle	24.3 ± 4.97	ON risk: 23.12 ± 4.57 Non-risk: 21.45 ± 3.57	0 (0%)
Blackburn (2024)	#ForYou? the impact of pro-ana TikTok content on body image dissatisfaction and internalisation of societal beauty standards	Australia	273	Women aged between 18–28 years sourced from the general population of TikTok users	between 18–28	not mentioned	0 (0%)
Unal (2024)	The Mediating Role of Attitudes Towards Eating in the Relationship Between Compulsive Instagram Use and Orthorexia in Adults.	Turkey	255	hospital staff and patients of a psychiatry outpatient clinic between June 2022 and May 2023	32.41 ± 7.101	N/A	128 (50.2%)
Bayram (2024)	Exploring the interplay between social media addiction, mindful eating, intuitive eating, orthorexia nervosa, and mental health in young adults	Turkey	303	university students between December 2023 and May 2024.	23.10 ± 4.31	22.40 ± 3.73	63 (20.46%)
Sener (2023)	Investigation of the Relationship Between Social Media Addiction and Orthorexia Nervosa in Adult Individuals Who Applied to Obesity Polyclinic	Turkey	174	obese individuals	most in 18–30 range	30.53 ± 5.10	57 (32.8%)
Silva (2023)	The body dissatisfaction role in the adoption of compulsive healthy eating behaviors	Portugal and Brazil	238	General population (specifically Instagram users)	Portuguese: 18–35Brazilian: 25–50	N/A	86 (36%)
Asil (2023)	The effect of social media use on orthorexia nervosa: a sample from Turkey	Turkey	2526	Adult social media users in Turkey	28.4 ± 10.3	23.6 ± 4.3	696 (27.6%)
Karniej (2023)	Orthorexia nervosa in gay men-the result of a spanish-polish eating disorders study	Poland & Spain	394	Gay men in Poland and Spain	38.08 ± 11.01	26.11 ± 4.71	394 (100%)
Levin (2023)	Examining orthorexia nervosa: Using latent profile analysis to explore potential diagnostic classification and subtypes in a non-clinical sample	Canada	333	Undergraduate students at a large urban university	20.9 ± 4.3	N/A	93 (28%)
Scheiber (2023)	Socio-cultural power of social media on orthorexia nervosa: An empirical investigation on the mediating role of thin-ideal and muscular internalization, appearance comparison, and body dissatisfaction	Germany and Austria	647	German-speaking sample of young men and women aged 18–30 years old	23.7 ± 4.7	23.7 ± 4.7	0 (0%)
Tarsitano (2022)	Symptoms of Orthorexia Nervosa are associated with time spent on social media: a web-based survey in an Italian population sample	Italy	4107	Italian population (Instagram users)	31 ± 9	24.4 ± 5	205 (5%)
Yurtdas-Depboylu (2022)	The association between social media addiction and orthorexia nervosa, eating attitudes, and body image among adolescents	Turkey	1232	High school adolescents in İzmir, Turkey	15.7 ± 1.19	N/A	520 (42.2%)
Gobin (2021)	The Effects of the COVID-19 Pandemic Lockdown on Eating, Body Image, and Social Media Habits Among Women With and Without Symptoms of Orthorexia Nervosa	Canada	143	Adult women aged 17–73 recruited during COVID-19 lockdown	25.85 ± 8.12	25.37 ± 7.45	0 (0%)
Yilmazel (2021)	Orthorexia tendency and social media addiction among candidate doctors and nurses	Turkey	969	Medical and nursing students	21.4 ± 3.2	70.8% normal BMI	350 (36.1%)
Turner (2017)	Instagram use is linked to increased symptoms of orthorexia nervosa	Global	680	Females aged 18–75 years	24.70 ± 7.87	22.14 ± 3.89	0 (0%)
Daniel (2025)	Relationships Between Orthorexia, Exercise Dependency, Body Image, and Decision-Making in University Undergraduates	USA	27 (but 26 pairs were analyzed)	Undergraduate students recruited from a university fitness center	20.4 ± 1.2	N/A	12 (44.44%)
Blažev (2025)	Biological, Behavioural, Psychological and Social Predictors of Orthorexia Nervosa Among Young Women in Croatia	Croatia	1209	Young women aged 18–21	N/A	N/A	0(0%)
Kus (2025)	From likes to obsessions: The mediating role of obsessive compulsive symptoms in the relationship between social media addiction and orthorexia nervosa	Turkey	321	Undergraduate university students	21.78 ± 2.29	21.51 ± 2.97	53(16.5%)
Biyikli (2024)	Evaluation of Orthorexia Nervosa Tendency and Social Media Addiction in University Students	Turkey	1153	University students	20.4 ± 2.0	N/A	418 (36.3%)
Christodoulou (2024)	Exploring the Link between Mindful Eating, Instagram Engagement, and Eating Disorders: A Focus on Orthorexia Nervosa	Greece	407	Greek adults (18–65 years)	N/A	N/A	31%
Hamurcu (2023)	The correlation between orthorexia nervosa and social media use in nursing students	Turkey	339	Nursing faculty students in Istanbul	20.74 ± 1.93	N/A	15.6%
Ulutaş (2025)	The type of social media is a greater influential factor for orthorexia nervosa than the duration: a cross-sectional study.	Turkey	892	Undergraduate university students	20.97 ± 2.26 years	Not reported but categories: Underweight 143 (16%) Normal 609 (68.3%) Overweight 113 (12.7%) Obese 27 (3%)	17.8%
Kovan (2025)	Social media use, eating attitudes, orthorexia nervosa and well-being: testing a moderated mediation model.	Turkey and Northern Cyprus	599	General adults	29.82 ± 9.39 years	Not reported	32.05%
Toğuç (2025)	Psychosocial effects of social media and nutrition information: an analysis of orthorexia nervosa and weight-related self-stigma in youth.	Turkey	651	University students (young adults)	20.85 ± 2.69 years	22.43 ± 3.63	17.8%
Ruiz-Lazaro (2025)	The Role of Social Media Addiction, Depression, Rumination and Disordered Eating Behaviours in Orthorexia Nervosa Symptoms.	Spain	321	Adults (18–49 years), mostly students (64.3% studying, 30.6% working)	23.06 ± 5.35 years	Not reported	66 (20.6%)
Thayuman (2025)	Nonattachment to self mediates the relationship between Instagram engagement and orthorexia nervosa amongst Australian university students.	Australia	299	Australian undergraduate university students	29.57 ± 11.91 years	Not reported	48 (16.1%)
Boutouis & Grant 2025	Problematic usage of the internet is associated with orthorexia nervosa tendency in adults	United States	290	General adult community sample recruited online via Prolific; aged 18 or over, US resident, English-speaking	37.40 + /- 12.61 years	Not reported	120 (42.3%)
Calpbinici 2025	Effect of social media use on the orthorexia nervosa tendency among pregnant women	Turkiye (Nevsehir)	200	Pregnant women at 12–40 weeks gestation attending a gynecology and obstetrics polyclinic; all were social media users	26.18 ± 5.55 years	26.89 ± 4.39 kg/m2	0 (0%)
Adiningsih et al. 2024	Effect of social media use on the orthorexia nervosa tendency among pregnant women	Indonesia (Pontianak)	66 quantitative + 10 interviewed	Medical students, 2020 and 2021 batches, Tanjungpura University, by proportionate stratified sampling; interviewees selected by convenience (3 highest and 2 lowest ON scorers per batch)	Not reported as a mean.	Not reported	25 (37.9%)

The studied populations mainly comprised young adults. Nearly all studies included more female participants than male, with five articles having no male participants [14,31,43,60,65,69]. Body Mass Index (BMI) among participants was mostly within the normal range [14,30,40,42,43,46,60–62]. (Table 1)

A variety of scales were used to assess ON among participants in the included studies. Most studies used the ORTO-15 Scale [14,29,31,47,55,60,64] or a modified version of the ORTO scale, the ORTO-11 [45,46,50,51,53–55,61,63,66]. The scales used to assess social media use varied greatly among studies, with some studies assessing social media use via custom, non-validated self-report measures [14,31,43,47,48,62,67,69] (Table 2).

**Table 2:** Key Findings Across Included Studies.

Author (Year)	Number (%) with ON Tendency	ON Tendency (Male %)	Social Media Scale (Mean Score)	Time spent on social media: Mean + SD	ON Scale (Mean Score)	Findings
Dobos (2024)	N/A	N/A	BSMAS (12.58 ± 4.87)	N/A	DOS (17.81 ± 5.61)	-Positive correlation between social media addiction and orthorexia nervosa-Higher social media addiction is linked to greater emotion regulation difficulties-Orthorexia nervosa was positively related to mindful eating and perfectionism-Emotion dysregulation was a key mediator between social media addiction and orthorexia nervosa-Perceived social media influence contributed to perfectionistic tendencies and maladaptive eating behaviors-Mindful eating can sometimes be linked to more rigid and restrictive eating habitsAdaptive perfectionism was positively associated with orthorexia nervosa
Awad (2024)	N/A	N/A	SMD (2.43 ± 2.34)	Less than 30 minutes/day: 28.1%30–60 minutes/day: 20.1%More than 1 hour/day: 51.8%	TOS (14.15 ± 5.97)	-Higher SMUD scores were significantly associated with higher ON scores (Beta = 0.50, p < 0.001).- Loneliness partially mediated the relationship between SMUD and ON, accounting for 16% of the indirect effect.-Using Instagram (Beta = 2.73, p = 0.007) and Tumblr (Beta = 3.22, p = 0.018) were significantly associated with higher ON scores.-Spending 30–60 minutes per day on social media apps (compared to less than 30 minutes) was significantly associated with higher ON scores (Beta = 2.33, p = 0.010).-Higher physical activity index was significantly associated with higher ON scores (Beta = 0.04, p = 0.007).
Staskiewicz-Bartecka (2024)	40 (28.2%) at risk29 (20.4%) with ON	N/A	N/A	Up to 1 hour: 11.3%1–2 hours: 30.3%2–3 hours: 26.8%Above 3 hours: 31.7%	DOS (Total Sample: 23.9 ± 6.7Polish Athletes: 23.1 ± 4.9Turkish Athletes: 21.4 ± 7.5Indian Athletes: 28.0 ± 5.5)	-49% of participants had an ON risk or presence-Higher social media use correlated with ON risk-Indian athletes had the highest ON prevalence-No significant correlation between BMI and ON risk-Athletes dissatisfied with body image were more likely to have ON-Social media use to seek diet information linked to ON risk
Kalender (2024)	N/A	N/A	SMA (2.84 ± 0.88)	N/A	ORTO-15 (2.86 ± 0.71)	-An elevation in participants’ perceptions of their healthy eating obsession (t = 5.170; p < .001) statistically raises their social–physique anxiety. -Increase in the perceptions of the participants’ obsession with healthy eating (t = 3.919; p < .001) increases their social media addiction statistically. -Increase in the perceptions of the participants’ social media addiction (t = 3.215; p < .001) statistically increases their social–physique anxiety
Piko (2024)	37.3% (cutoff score of 35)64.8% (cutoff score of 40)	0	BSMAS (ON risk group: 14.35 ± 5.45; Non risk group: 12.88 ± 5.07)	N/A	ORTO-15 (37.81 ± 6.74)	- Eating disorder attitudes (drive for thinness, ineffectiveness and maturity fears), regular sporting activity and social media addiction proved to be relevant predictors of these women’s ON tendency (with drive for thinness being the strongest contributor).-BMI (positively) and proactive coping (negatively) were correlated with the ORTO-15 score. (became nonsignificant in the multivariate analysis).-The prevalence of ON risk was approximately 38%.
Blackburn (2024)	N/A	0	self-reported	-Low TikTok use: Healthy DE(10.61 ± 2.43), Restrictive DE(14.76 ± 14.77)-Moderate TikTok use: Healthy DE(10.54 ± 2.79), Restrictive DE(15.53 ± 13.15)-High TikTok use: Healthy DE(10.56 ± 2.85), Restricitve DE(18.16 ± 13.33)-Extreme: Healthy DE(11.09 ± 2.71), Restrictive DE(19.09 ± 14.31)- < 30 minutes: 13.6%-30 minutes to 1 hour: 26.4%-1–2 hours: 31.9%-2–3 hours: 15.8%-3–4 hours: 10.6%-4–5 hours: 1.1%-Over 5 hours: 0.7%	ORTO-15 (N/A)	-64% of participants reported being exposed to disordered eating content via their For You page.- Only 15% of participants had not been exposed to any negative content themes.-43% of the participant sample were frequently exposed to fitness and sports-related content-There was no statistically significant difference at the p < .05 level in ORTO-15 scores for the four TikTok usage groups-EAT-26 scores across the four TikTok usage groups also yielded a non-significant resultExperimental Findings:-The body image satisfaction scores for women in both conditions(cases and controls) decreased from pre-manipulation to post-manipulation(time).- Participants in the experimental condition reported a greater decrease in body image satisfaction than women in the control condition- The internalization scores for women in the control group decreased from pre-manipulation to post-manipulation, while in the experimental group, internalization scores increased
Unal (2024)	N/A	40.082 ± 12.714	SMA (75.482 ± 31.840)	9.08 h ± 4.203	Orthorexia Nervosa Inventory (39.822 ± 11.983)	-There were no significant differences in scores between sexes.-Total orthorexia scores were positively correlated with eating attitudes, total scores of compulsive use of social media, occupation subdimension of compulsive use of social media, mood modification subdimension of compulsive use of social media, relapse subdimension of compulsive use of social media and conflict subdimension of compulsive use of social media (r = 0.332, p < 0.001).-only the eating attitude significantly predicted the behaviors subfactor of orthorexia.- Conflict(Social Media Addiction Scale subfactor) and eating attitude predicted the impairments subfactor of orthorexia.-eating attitude predicted the emotions subfactor of orthorexia.-significant and positive relationship between compulsive use of social media and orthorexia.-a positive and significant relationship between compulsive use of social media and eating attitude
Bayram (2024)	Risk of ON 205	N/A	SMA (N/A)	-Not addictive (41–73 scores): 78 (25.7%)-Less addictive (74–106 scores): 102 (33.7%)-Moderately addictive (107–139 scores): 84 (27.7%)-Highly addictive (140–172 scores): 28 (9.2%)-Very highly addictive (173–205 scores): 11 (3.6%)	ORTO-11 (N/A)	-Participants at risk of orthorexia nervosa, as classified by ORTO-11, exhibited significantly higher scores on the IES-2 and SMAS compared to those without risk.-The SMAS exhibited a weak positive correlation with depression, anxiety, and IES-2 scores.- SMAS displayed a negative correlation with ORTO-11.-a moderate correlation was observed between SMAS and stress and MEQ.-ORTO-11 demonstrated a positive moderate correlation with IES-2 scores, however, a negative moderate correlation with MEQ.- all predictors(Age, Sex, IES-2, MEQ, and SMAS) were significantly associated with the risk of ON.
Sener (2023)	N/A	N/A	SMA (46.47 ± 14.78)	N/A	ORTO-11 (28.33 ± 4.61)	-Higher ON prevalence in obese individuals.-Significant negative correlation between ORTO-11 score and social media addiction (especially “virtual communication” sub-dimension).-Obese participants had a higher virtual communication score compared to overweight individuals.-Younger participants (18–30) had higher social media addiction scores.-No significant relationship between BMI and ON score.
Silva (2023)	N/A	N/A	Instagram use scale (N/A)	N/A	ORTO-15 (N/A)	-Instagram use (IU) and fitness content (FT) do not directly increase Body Dissatisfaction (BD) or ON.-Internalization (IN), upward (UP), and downward (DO) social comparisons increase BD, which mediates the development of ON.-Perfectionism (PF) and obsessive-compulsive (OC) traits increase BD and ON.-BD significantly mediates the relationship between IN, UP, DO, PF, and ON.
Asil (2023)	56.1% (1416)	360 (25.4%)	SMEB (46.3 ± 21.3)	167.4 ± 141.2 minutes/day	ORTO-11 (25.6 ± 5.4)	-Increased ON Tendency with Age and BMI: ON tendency increased with age (OR: 1.014, 95% CI: 1.003–1.024) and BMI (OR: 1.037, 95% CI: 1.013–1.062).-Social Media Influence: Following health-related pages (e.g., healthy nutrition and sports/exercise recommendations) increased the risk of ON (OR: 2.441 and 1.680, respectively).-Gender Difference: Women had a higher risk of ON tendency compared to men (OR: 1.285).-Time Spent on Social Media: Participants without ON tendency spent more time on social media (172.2 ± 119.9 minutes/day) compared to those with ON tendency (161.1 ± 117.9 minutes/day).
Karniej (2023)	131 (33.25%)	100%	N/A	N/A	ORTO-15 (N/A)	-Grindr usage was significantly associated with higher risk of ON (OR = 5.312, p < 0.001).-Instagram use was associated with a lower risk of ON (OR = 0.479, p = 0.008).-Lower BMI was associated with higher risk of ON (OR = 0.895, p < 0.001).-Daily use of PrEP was associated with lower risk of ON (OR = 0.142, p = 0.014).-Occasional use of PrEP was associated with increased risk of ON (OR = 4.667, p = 0.028).-Being in an informal relationship was associated with higher risk of ON (OR = 2.138, p = 0.007)
Levin (2023)	74 (22.2%)	N/A	N/A	N/A	EHQ (Knowledge/Behaviors: 14.9 ± 4.9Problems: 14.2 ± 4.6Feelings: 10.9 ± 2.6Total: 40.1 ± 10.0)	-3 ON subtypes were identified:1-ON/ED Combined2-ON/ED Combined (without weight/shape concerns)3-ON only-ON was found to have stronger overlap with ED symptoms than with OCD, OCPD, or health anxiety-Higher social media use (viewing/sharing healthy eating content) is linked to ON symptoms, especially in the ON/ED Without Weight/Shape Concerns group.-ON Only group is more likely to be male, while ON/ED Combined is associated with higher BMI.
Scheiber (2023)	N/A	N/A	social media measured using three items based on Schaefer, Terlutter, & Diehl (2019) adapted to the social media context. (4.3 ± 1.7)	N/A	DOS (2.7 ± 1.4)	-Involvement in health and fitness accounts on social media was positively and directly associated with ON tendencies-involvement with health and fitness accounts on social media was also positively related to thin-ideal internalization.- thin-ideal internalization was also positively related to ON tendencies- positive relationship between involvement and muscular internalization and muscular internalization was also positively related to ON tendencies-involvement was also positively associated with appearance comparison however, appearance comparison was not positively related to ON tendencies-involvement was not positively associated with body dissatisfaction and body dissatisfaction was also not positively related to ON-Appearance comparison was positively associated with body dissatisfaction- Thin-ideal internalization was positively related to body dissatisfaction-Muscular internalization was not significantly related to body dissatisfaction
Tarsitano (2022)	28.5% (1170 participants with I-DOS score ≥ 35)	N/A	self reported	<15 min: 21.8% with ON>60 min: 31% with ON	DOS (30.6 ± 6)	Longer time spent on social media (especially >60 min/day) is associated with higher prevalence of ON.Instagram is the most used social media platform (93.2%).28.5% of participants had ON (I-DOS score ≥ 35).Participants who spent >60 min/day on social media had a 31% prevalence of ON, compared to 21.8% for those who spent <15 min/day.
Yurtdas-Depboylu (2022)	27.9% (344 participants with ORTO-11 score ≤ 27)	26.70%	SMASA (19.2 ± 6.98)	<1 h: 8.7%1–3 h: 49.7%4–6 h: 34.0% ≥ 7 h: 7.6%	ORTO-11 (30.6 ± 5.53)	-Social media addiction was significantly associated with a greater risk of eating behavior disorders (OR: 1.07, 95% CI: 1.04–1.10) and ON tendency (OR: 1.02, 95% CI: 1.00–1.04).-Adolescents who frequently read nutrition-related posts on social media had a higher likelihood of eating behavior disorder risk (OR: 6.77, 95% CI: 3.84–11.94) and ON tendency (OR: 5.49, 95% CI: 3.39–8.88).-Better body image was associated with lower social media addiction scores (β = −0.268, p < 0.001).
Gobin (2021)	median split (around 50%)	N/A	N/A	-Participants high on ON symptoms: 52.2 ± 49.2 minutes per day-Participants low on ON symptoms: 36.6 ± 28.8 minutes per day	EHQ (N/A)	-Women high on ON symptoms reported more negative eating behaviors (eating more, thinking about food more).-High ON participants felt greater pressure to diet, lose weight, and exercise due to social media.-Increased food consumption and weight gain were more common in high ON participants.-Perceived pressure from social media to lose weight was significantly higher in the high ON group.
Yilmazel (2021)	62.20%	60.10%	SMA (93.2 ± 31.5)	N/A	ORTO‐15 (N/A)	-78.8% of the participants were social media addicts.(prevalence was higher among candidate nurses than candidate doctors)-62.2% of the participants were determined to have an orthorexia tendency.(no significant difference between age groups in terms of orthorexia tendency)-no significant difference in terms of orthorexia tendency according to gender(M 60.1%, F 66%)-the ratio of ON tendency was 54.5% among candidate nurses, whereas it was 73.0% among candidate doctors.- the ratio of ON tendency was 68.5% for those with a family income of 1,000 USD or above and was significantly higher than other income groups-Orthorexia tendency was not significantly different in terms of chronic problems, health perception, physical activity, weight satisfaction, and BMI.-Orthorexia tendency was 1.68 times higher in those taking no vitamin supplements, 1.73 times higher in non‐dieters, and 1.37 times higher in social media addicts.
Turner (2017)	90.6% cut-off score of <4049.0% a cut-off score of <35.	0	N/A	Median time users spend on this channel per day:-Instgram: 15–30 min-Facebook: 15–30 min-Twitter: < 15 min -Pintrest: 15–30 min-Google + : < 15 min-Tumblr: 15–30 min-LinkedIn: < 15 min	ORTO-15 (34.33 ± 4.04)	-All but one participant used at least one social media channel(intgram being most popular, while google+ is least)-80% of participants who used Instagram ranked food as 1st or 2nd most frequent image category appearing on their Instagram feed.-No relationship was found between ORTO-15 scores and number of food types eaten, number of social media channels used, or BMI.-no significant differences in ORTO-15 score between diet types as determined by one-way ANOVA.-a negative correlation between ORTO-15 score and Instagram use.-No significant relationship was found between ORTO-15 score and other social media use, except Twitter which showed a small positive correlation
Daniel (2025)	N/A	N/A	Investigator-designed scale (28.9 ± 6.5)	N/A	TOS (37.3 ± 10.1)	-Social media engagement (fitness-themed) was significantly correlated with Exercise Dependence (r = 0.58, p = 0.002) and Body Dysmorphia severity (r = 0.42, p = 0.03). -Exercise Dependence was significantly correlated with Orthorexia Nervosa (r = 0.40, p = 0.04) and overall ON symptoms (r = 0.53, p = 0.005). -Body Dysmorphia symptoms were significantly correlated with Orthorexia Nervosa (r = 0.48, p = 0.01) and overall ON symptoms (r = 0.45, p = 0.02). -No significant correlations were found between any of these measures (ON, exercise dependence, body dysmorphia, social media use) and decision-making performance on the Iowa Gambling Task.
Zagreb (2025)	N/A	N/A	Custom items assessing health-related social media engagement (N/A)	<15 min: 36.5% 15–30 min: 30.8% 30–60 min: 14.8% 1–2 hours: 5.8% 2–3 hours: 1.5% 3 hours: 0.7% None: 10.0%	DOS(N/A)	-Hierarchical regression explained 36% of variance in ON symptoms. -Social factors contributed most (10.6%), followed by psychological (9.9%) and behavioral factors (8.7%). -Significant predictors of higher ON symptoms: restrictive dieting, obsessive-compulsive symptoms, narcissistic perfectionism, body dissatisfaction, internalization of thin/muscular ideals, media pressures, and higher engagement with health-related social media content (more time spent and more accounts followed). -Number of social media platforms followed was not a significant predictor. -Media pressure (but not family or peer pressure) significantly predicted ON.
Kus (2025)	N/A	N/A	SMAS-AF(51.96 ± 13.16)	0-1 hours: 3(0.9) 2-4 hours: 83(25.9) 5-7 hours: 162(50.5) >8 hours: 73(22.7)	ORTO-11(27.74 ± 4.85)	-Significant positive correlation between social media addiction and obsessive-compulsive symptoms (OCS). -Significant negative correlation between ORTO-11 scores and OCS (i.e., lower ORTO-11 score = higher ON tendency). -Mediation analysis showed that social media addiction indirectly increases ON tendency through obsessive-compulsive symptoms (full mediation). -OCS explained 13% of variance in ON. -Direct effect of social media addiction on ON was nonsignificant.
Biyikli (2024)	24%	N/A	BSMAS(16.9 ± 4.6)	N/A	ORTO-11(N/A)	-ON tendency was significantly higher in females than males (p < 0.05). -ON tendency increased significantly with higher BMI (p < 0.05). -Significant negative correlation between ORTO-11 scores and BSMAS scores (r = –0.058, p = 0.048), meaning higher social media addiction was associated with higher ON tendency (lower ORTO-11 score). -Social media addiction was higher in females but not significantly different from males.
Christodoulou (2024)	N/A	N/A	Instagram use (daily time categories)(N/A)	15.5% < 1h 35.2% 1–3h 46.5% > 3h daily	ORTO-R(N/A)	-Higher mindful eating (MES-16) correlated with lower ON (ORTO-R) and eating disorder scores. -Higher Instagram use (>3h/day) associated with higher ORTO-R scores. -Women with higher ORTO-R scores had nearly 3x higher odds of using Instagram > 3h/day.
Hamurcu (2023)	103 (30.38%)	N/A	SMUIS(27.53 ± 8.77)	3.42 ± 1.91 hours/day	ORTO-11(27.98 ± 4.92)	-A weak, significant negative correlation was found between ORTO-11 scores and SMUIS total score (r = -0.136, p = 0.01), the “Integration into Social Routines” sub-dimension (r = -0.138, p = 0.01), and daily duration of social media use (r = -0.11, p = 0.04). -No significant difference in ON tendency based on the specific social media platform used (e.g., Instagram, Facebook). -Students who reported their decisions were not influenced by social media had significantly higher ORTO-11 scores (lower ON tendency) than those who were influenced (p < 0.001).
Ulutaş (2025)	205 (23%)	N/A	30.66 ± 8.84	396.78 ± 270.89 min/day	27.44 ± 5.25	- ON tendency associated with use of visual-based platforms: Instagram (p = 0.006), Pinterest (p = 0.001), TikTok (p = 0.001). - No significant correlation between total SM duration and ON. - Higher SMIU scores correlated with higher ON tendency (r = -0.151, p < 0.001). - Following non-expert sources (influencers, artists, news accounts) for nutrition info linked to higher ON. - ON also associated with: being single, higher BMI, studying Nutrition & Dietetics, receiving medical nutrition therapy, recent dieting, shopping influenced by SM, and higher physical activity.
Kovan (2025)	Not reported (cut-off is 24)	N/A	20.88 ± 3.01	<2h (11.85%) 3–5h (63.10%) ≥6h (25.04%)	28.05 ± 4.07	- A Pearson correlation showed no statistically significant direct association between social media use and orthorexia nervosa (ORTO-11) scores (r = -0.05, p > 0.01). - However, ON significantly moderated the indirect relationship between social media use and well-being through eating attitudes. The interaction between social media use and ON levels negatively predicted eating attitudes (β = -0.24, p < 0.001). - Contrary to expectations, the conditional effect of social media use on well-being was significant only among participants with low levels of orthorexia nervosa, not among those with moderate or high levels.
Toğuç (2025)	Not reported (High-risk cutoff is score >72)	N/A	Not reported But there is Social Media Addiction Score Female M 17.93 & Male M 16.87	N/A	Total sample mean not provided 43.03 ± 12.71 (Women) 44.03 ± 14.56 (Men)	- Social media addiction score was positively correlated with total ON score (r = 0.150, p < 0.001). - Participants classified with social media addiction had significantly higher total ON scores than those without (p = 0.001). - In a multivariate model predicting ON, weight self-stigma (β = 0.385, p < 0.001) and e-healthy diet literacy (β = 0.176, p < 0.001) were significant predictors, but social media addiction was not (β = 0.039, p = 0.277). - Social media addiction was a significant predictor of weight self-stigma (β = 0.166, p < 0.001) in a separate model.
Ruiz-Lazaro (2025)	150 participants (46.7%) at high risk for ON (ORTO-15 score < 40)	N/A	13.10 ± 6.79	N/A	39.53 ± 6.41	- Positive association between social media addiction and ON symptoms (r = −0.36, p < 0.01; note: ON score is inverse). - Higher ED-specific rumination (both brooding and reflective) associated with higher ON symptoms. - Higher depressive symptoms associated with lower ON symptoms. - Higher eating disorder symptoms associated with higher ON symptoms. - Path analysis indicated indirect effects of ED-brooding rumination on ON via social media addiction and ED symptoms.
Thayuman (2025)	Not reported	N/A	36.02 ± 10.31	Not reported (there is an intensity scale used of Mean Score: 36.02 ± 10.31)	19.34 ± 4.31	- Instagram engagement positively correlated with ON symptoms (r = 0.279, p < 0.001). - Nonattachment to self (NTS) negatively correlated with both Instagram engagement and ON. - NTS partially mediated the relationship between Instagram engagement and ON (indirect effect B = 0.016, 95% CI [0.004, 0.030]). - The direct effect of Instagram on ON remained significant after mediation (B = 0.10, p < 0.001).
Boutouis & Grant 2025	Not reported	Not reported	No social media scale was administered. ISAAQ-10 total problematic internet use: 18.48 + /- 9.29 (alpha = 0.903); 12.8% met the PUI cutoff of 30/50. Social networking captured only as one ISAAQ part B frequency item	Not reported	ORTO-R 14.52 + /- 4.95 (alpha = 0.809)	-ISAAQ-10 correlated with ORTO-R (r = .411, p < .001) and predicted ORTO-R after adjusting for age, gender and eating disorder diagnosis (beta = .375, p < .001; model R2 = .203). -Higher ORTO-R scores were found with frequent online shopping (p = .023), cyberchondria (p = .042), researching healthy food online (p < .001, partial eta2 = .169, the largest effect in the study) and any cyberbullying perpetration (p = .003). -Critically, ON tendency did NOT differ by social networking frequency: 14.34 + /- 4.82 versus 14.70 + /- 5.06, F(1,278) = .054, p = .816, partial eta2 = .000. -The authors state the direction of the association is unknown
Calpbinici 2025	Not reported as a count	N/A	SMUIS total 29.37 + /- 10.03 (Turkish version alpha = 0.87; no cutoff). Subscales: Social Integration and Emotional Attachment 14.97 + /- 6.49; Integration with Social Routines 14.40 + /- 4.49	Not reported as a mean	ORTO-11 29.18 + /- 4.52 (alpha = 0.64 in this sample)	-SMUIS total was inversely correlated with ORTO-11 (r = -0.145, p = 0.040). -Because the ORTO-11 is reverse-scored, this means greater social media integration accompanied greater ON tendency, giving a harmonized r of +0.145. -Subscales: Social Integration and Emotional Attachment r = -0.178, p = 0.012; Integration with Social Routines r = -0.068, p = 0.341 (not significant). -Hierarchical regression predictors of ON tendency: SMUIS beta = 0.829, age beta = -0.183, place of residence beta = 0.181, intended pregnancy beta = 0.140, attention to food intake beta = 0.139, BMI beta = 0.151 (all p < 0.05). -BMI category was associated with ORTO-11 score (p = 0.045).
Adiningsih et al. 2024	37 (56.1%) with ON indication; 29 (43.9%) without. ORTO-15 cutoff below 40	Not reported	No social media instrument was administered. Social media addressed only in the qualitative interviews	Not reported	Not reported	=No significant quantitative association between ON and BMI (Spearman p = 0.139) or diet (p = 0.105); the eating-pattern analysis was invalid because all 66 students were classified as normal, leaving zero variance. -Qualitative: both high and low ON scorers described social media influencing their food choices, using it to seek healthy-eating inspiration, and being driven by diet and fashion pages. =The authors attribute ON in this group to nutrition knowledge and a desire to avoid illness rather than to any measured exposure

Most studies demonstrated a positive association between social media use and ON. a negative correlation was observed between ORTO-11 score and social media, suggesting that greater social media use was associated with more heightened orthorexia tendencies [45,51,61]. Likewise, a strong positive relationship was observed between compulsive social media use and orthorexia nervosa [44]. In a study done on healthcare workers, orthorexia tendency was found to be 1.37 times higher in chronic social media users [64].

Several studies examined the association between time spent on social media and ON. Awad et al. [59] demonstrated that a higher ON score was associated with more time spent on social media apps (> 30 minutes). Similarly, Tarsitano et al. [55] showed that the prevalence of ON increases with more than 60 minutes of daily social media usage. Stronger ON-related symptoms were seen in higher Instagram use (>3 hours/day) [54]. In another paper, participants high on ON tendencies reported spending more time on social media (52.2 ± 49.2 minutes per day) compared to participants low on ON tendencies (36.6 ± 28.8 minutes per day) [69]. However one investigation reported an opposite trend, where participants without ON tendencies spent more time on social media (172.2 ± 119.9 minutes/day) compared to those with ON tendencies (161.1 ± 117.9 minutes/day) [46]. A study exploring differences in ORTO-15 scores among four TikTok usage groups studied (low, moderate, high, and extreme)found no significant statistical differences [31] (Table 2).

The role of specific social media platforms and content type was found to have a significant effect on ON tendencies. Viewing and sharing health related material was associated with higher ON risk [30,43,46,48,63,67]. For instance, female football players who used social media to find diet information were linked to a higher risk of ON [43]. Platform-specific investigations yielded mixed findings. Instagram and Tumblr usage was correlated with a higher ON score [59]. Turner and Lefevre [14] similarly found a negative correlation between Instagram and ORTO-15 scores, however Twitter showed a small positive association. In a population of gay men, Instagram usage was found to be linked with a lower risk of ON and instead the use of Grinder was associated with higher risk of ON [47]. Other studies found no significant association between platform type and ON tendencies. Among nursing students, no significant difference was found in ON tendency between different social media platforms(e.g., Facebook, and Instagram) [53]. The number of social media platforms followed was also not a significant predictor of ON [65]. (Table 2)

A consistent theme across the studies was that psychological mechanisms mediate the relationship between ON and social media. For example, Body dissatisfaction was found to be a key mediator. Silva et al. indicated that Instagram use and fitness content don’t directly increase ON and body dissatisfaction; rather, social comparison increases body dissatisfaction that contributes to ON development [29]. Similarly, engaging in fitness themed content on social media was linked to heightened body dysmorphia symptoms, which showed a positive correlation with ON symptoms [41]. Among young women, body dissatisfaction was also positively associated with ON risk [43,67]. In contrast, Scheiber et al. [30] found no association between body dissatisfaction and ON. Emotional and personality traits were also prominent factors. Dobos et al. [40] found that emotional dysregulation and perfectionism linked ON and problematic social media use. Similarly, Silva et al. [29] reported perfectionism and obsessive-compulsive traits as mediators for ON and body dissatisfaction. Kalender and Kisi [42] further noted that heightened obsession with healthy eating and social media use contributed to social-physique anxiety. Moreover, no direct effect of social media on ON was found in one study, however obsessive compulsive symptoms were found to mediate this relationship [50]. Finally, overlaps between ON and other psychopathologies were observed. Levin et al. [48] identified ON subtypes that overlapped most strongly with eating disorder features, while Piko et al. [60] found that eating disorder-related traits, such as drive for thinness, were the strongest predictors of ON tendency. Both obsessive-compulsive symptoms and narcissistic perfectionism were associated with higher ON symptoms [67]. (Table 2)

### Meta-analysis results

Of the 33 included studies, 22 reported a usable zero-order correlation between a continuous social media measure and a continuous orthorexia score and were pooled using Fisher’s z transformation under a random-effects (REML) model, with reverse-scored ORTO-11/ORTO-15 coefficients recoded so that a positive value consistently denotes greater social media use accompanying greater orthorexia (total N = 10,868; Table 3). The pooled correlation was r = 0.20 (95% CI, 0.15 to 0.25), indicating a small positive association between social media use and orthorexia nervosa symptoms (Fig 2). Between-study heterogeneity was substantial: Q(21) = 140.77 (p < .001), I² = 84.3%, τ² = 0.0112 (τ = 0.106). The 95% prediction interval was −0.02 to 0.40, so the pooled value should be read as the centre of a distribution of effects rather than the correlation to be expected in any given setting, and the evidence remains compatible with a negligible association in some populations even though all 22 harmonized coefficients were positive.

**Table 3:** Studies Contributing to the Correlational Meta-Analysis.

Author (Year)	ON instrument	Scoring	Social media measure	Construct	r (harmonized)	n
Unal & Kocatepe Avci 2024	ONI	Normal	SM Addiction Scale	Addiction	+0.332	255
Toguc & Hokelek 2025	ONI	Normal	BSMAS	Addiction	+0.150	651
Ruiz-Lazaro et al. 2025	ORTO-15	Reverse	SNAS	Addiction	+0.360	321
Ozkefeli Hamurcu & Yilmaz 2023	ORTO-11	Reverse	SMUIS	Intensity	+0.136	339
Thayuman et al. 2025	ORTO-R	Normal	MFIS (Instagram)	Intensity	+0.279	299
Kovan & Yildirim 2025	ORTO-11	Reverse	SMUPS	Purpose	+0.050	599
Sener & Ozkaya 2023	ORTO-11	Reverse	SM Addiction Adult Form	Addiction	+0.141	174
Dobos et al. 2024	DOS-10	Normal	BSMAS	Addiction	+0.080	551
Awad et al. 2024	TOS (OrNe)	Normal	SMD-9	Addiction	+0.230	363
Kalender & Kisi 2024	Healthy Eating Obsession	Reverse	BSMAS	Addiction	+0.193	400
Piko et al. 2024	ORTO-15	Reverse	BSMAS	Addiction	+0.300	310
Toptas Biyikli & Biyikli 2024	ORTO-11	Reverse	BSMAS	Addiction	+0.058	1153
Kus et al. 2025	ORTO-11	Reverse	SM Addiction Adult Form	Addiction	+0.097	321
Usta Ulutas & Okan Bakir 2025	ORTO-11	Reverse	SMUIS	Intensity	+0.151	892
Scheiber et al. 2023	DOS (German)	Normal	3-item SM scale	Other	+0.327	647
Blazev 2025	DOS	Normal	Custom health-SM items	Other	+0.300	1209
Daniel & Gendle 2025	TOS	Normal	Investigator fitness-SM scale	Platform	+0.330	26
Boutouis & Grant 2025	ORTO-R	Normal	ISAAQ-10 (total PUI)	Addiction	+0.411	290
Yurtdas-Depboylu et al. 2022	ORTO-11	Reverse	SMASA	Addiction	+0.065	1232
Bayram et al. 2024	ORTO-11	Reverse	SMAS	Addiction	+0.104	303
Calpbinici 2025	ORTO-11 / HEOS	Reverse	SMUIS	Intensity	+0.145	200
Levin et al. 2023	EHQ	Normal	Content viewing and sharing	Purpose	+0.223	333

Direction refers to the scoring of the orthorexia instrument. Coefficients from reverse-scored instruments were sign-flipped so that a positive value denotes greater social media use accompanying greater orthorexia. For Daniel & Gendle 2025, 27 participants were enrolled and the correlation is based on 26 complete pairs; 26 is the value carried into the meta-analysis. Total N = 10,868.

**Fig 2:**
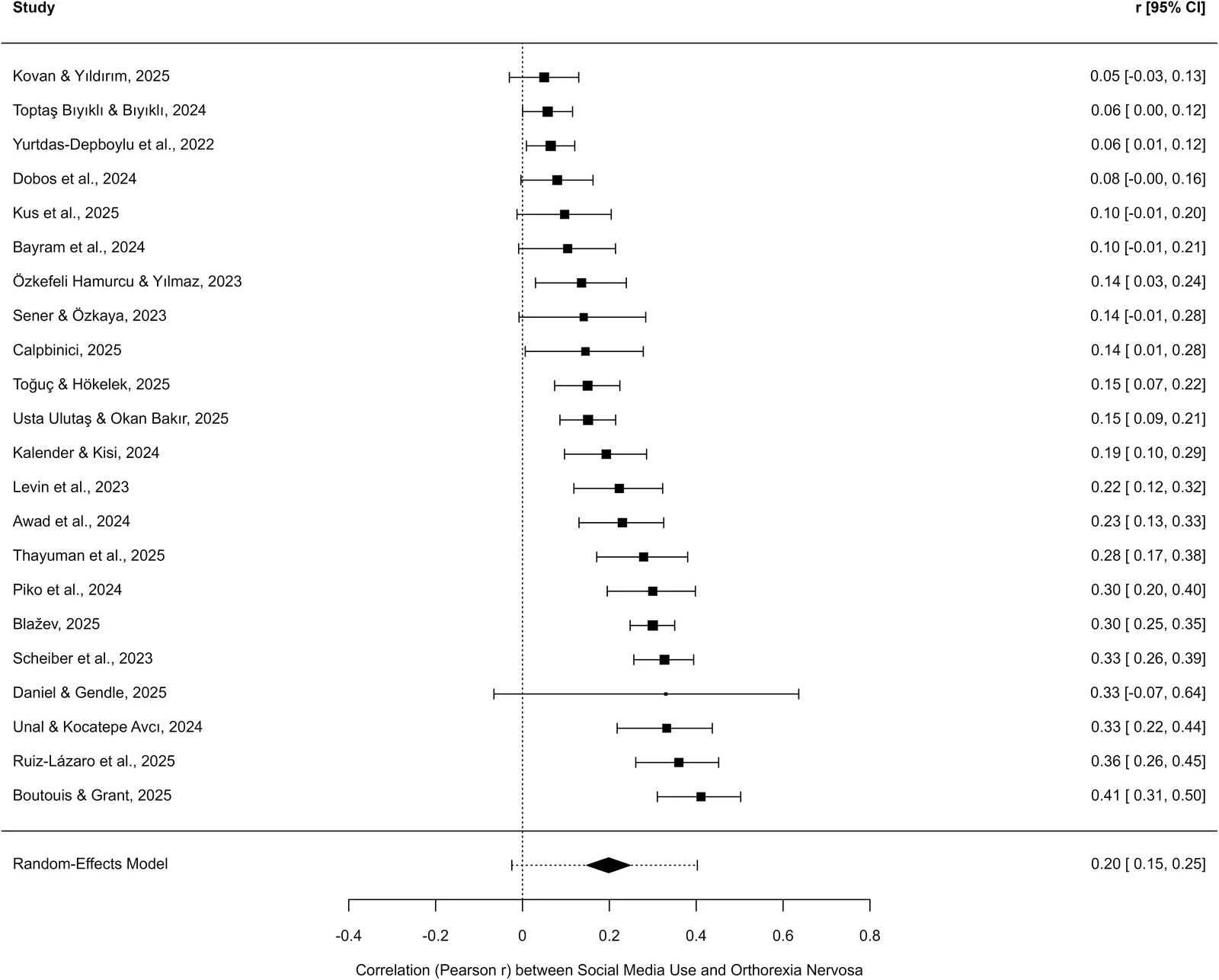
Pooled correlation between social media use and orthorexia nervosa symptoms.

**Moderator analyses.** Subgroup and meta-regression results are presented in Table 4. A meta-regression of study-level effect size on publication year found no significant trend over time (b = 0.027, p = .35; Fig 3), indicating the association has not systematically strengthened or weakened across the study period. Subgroup analysis by scale-scoring direction showed a stronger pooled association among studies using normally-scored instruments (k = 10, r = 0.26, 95% CI 0.19–0.33) than among those using reverse-scored ORTO instruments (k = 12, r = 0.15, 95% CI 0.08–0.21; F(1,20) = 7.08, p = .015), accounting for approximately 26.7% of the observed heterogeneity. This is best read as a measurement artefact rather than a substantive effect modifier: the reverse-scored ORTO family, criticized for capturing healthy eating as much as pathology, attenuates the association. None of the four remaining pre-specified moderators explained a significant share of the variance: social media construct (F(4,17) = 0.94, p = 0.47, R² = 3.5%), orthorexia instrument family (F(4,17) = 0.35, p = 0.84, R² = 0%), correlation coefficient type (F(1,20) = 0.04, p = 0.84; Pearson k = 18, r = 0.20; Spearman k = 4, r = 0.19) and sample size as log n (b = −0.057, p = 0.14, R² = 7.2%). Notably, the pooled correlation did not differ materially between ORTO-family instruments (k = 14, r = 0.18, 95% CI 0.11 to 0.24) and the non-ORTO instruments DOS, TOS, ONI and EHQ (r = 0.22 to 0.24 across four subgroups), indicating that the attenuation tracks scoring direction rather than the ORTO family as such.

**Table 4:** Subgroup and Meta-Regression Results for the Pooled Correlation.

Moderator	Level	k	r or b	95% CI	Test	R-squared
Scale-scoring direction	Normally scored	10	0.26	0.19 to 0.33	F(1,20) = 7.08, p = 0.015	26.7%
	Reverse-scored ORTO	12	0.15	0.08 to 0.21		
ON instrument family	ORTO family	14	0.18	0.11 to 0.24	F(4,17) = 0.35, p = 0.84	0%
	DOS	3	0.24	0.10 to 0.37		
	TOS	2	0.24	0.03 to 0.45		
	ONI	2	0.24	0.06 to 0.40		
	EHQ	1	0.22	-0.04 to 0.45		
Social media construct	Addiction	13	0.19	0.13 to 0.26	F(4,17) = 0.94, p = 0.47	3.5%
	Intensity	4	0.18	0.06 to 0.29		
	Purpose	2	0.13	-0.04 to 0.30		
	Platform	1	0.33	-0.15 to 0.68		
	Other	2	0.31	0.16 to 0.45		
Correlation type	Pearson	18	0.20	0.14 to 0.26	F(1,20) = 0.04, p = 0.84	0%
	Spearman	4	0.19	0.06 to 0.32		
Publication year	Meta-regression	22	b = 0.027	-0.031 to 0.085	p = 0.35 (perm. p = 0.37)	0%
Sample size (log n)	Meta-regression	22	b = -0.057	-0.135 to 0.021	p = 0.14	7.2%

All models were fitted with small-sample adjusted (Knapp-Hartung) standard errors on a common between-study variance. R-squared is the proportion of between-study heterogeneity explained. Scale-scoring direction was the only moderator reaching significance.

**Fig 3:**
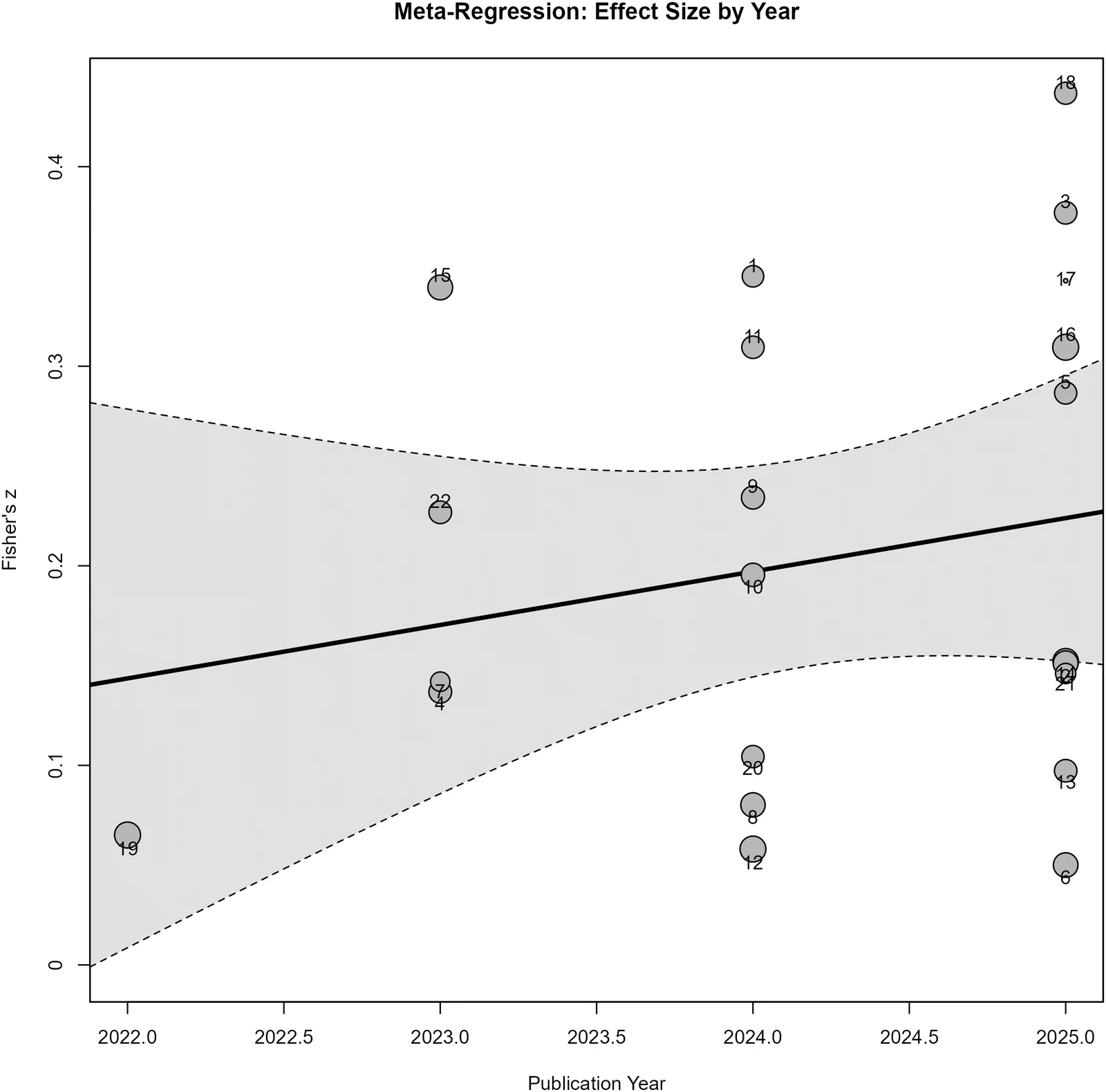
Meta-regression of the association between social media use and orthorexia nervosa symptoms by publication year.

**Robustness and influence diagnostics.** Leave-one-out analysis showed the pooled estimate was stable, ranging only from r = 0.19 to r = 0.21 across all 22 re-estimations, with every interval excluding zero (Fig 4). Influence diagnostics (studentized residuals, DFFITS, Cook’s distance, covariance ratio, hat values) identified one comparatively high-influence study (Boutouis & Grant, 2025; see Table 1) that nonetheless remained within an acceptable range (Fig 5), and a Baujat plot confirmed that most studies clustered near the origin, indicating limited individual influence on the summary estimate (Fig 6). Seven pre-specified sensitivity analyses — excluding, in turn, Spearman-derived coefficients, composite measures, the single very small sample (n = 26 analyzed of 27 enrolled), studies whose effect sign had to be inferred, content-specific measures, the pregnancy sample (Calpinci 2025), and a model restricted to social-media-addiction-specific measures only — left the pooled correlation within a narrow band of r = 0.18 to r = 0.20 in every scenario (Fig 7). An eighth model excluding the single study rated at high risk of bias on the Newcastle-Ottawa Scale (Daniel & Gendle 2025) gave a pooled correlation of r = 0.20 (95% CI 0.15 to 0.24; k = 21, I² = 85.0%), essentially unchanged from the primary estimate. Taken together, the pooled estimate was not materially altered by any single pre-specified analytic decision.

**Fig 4:**
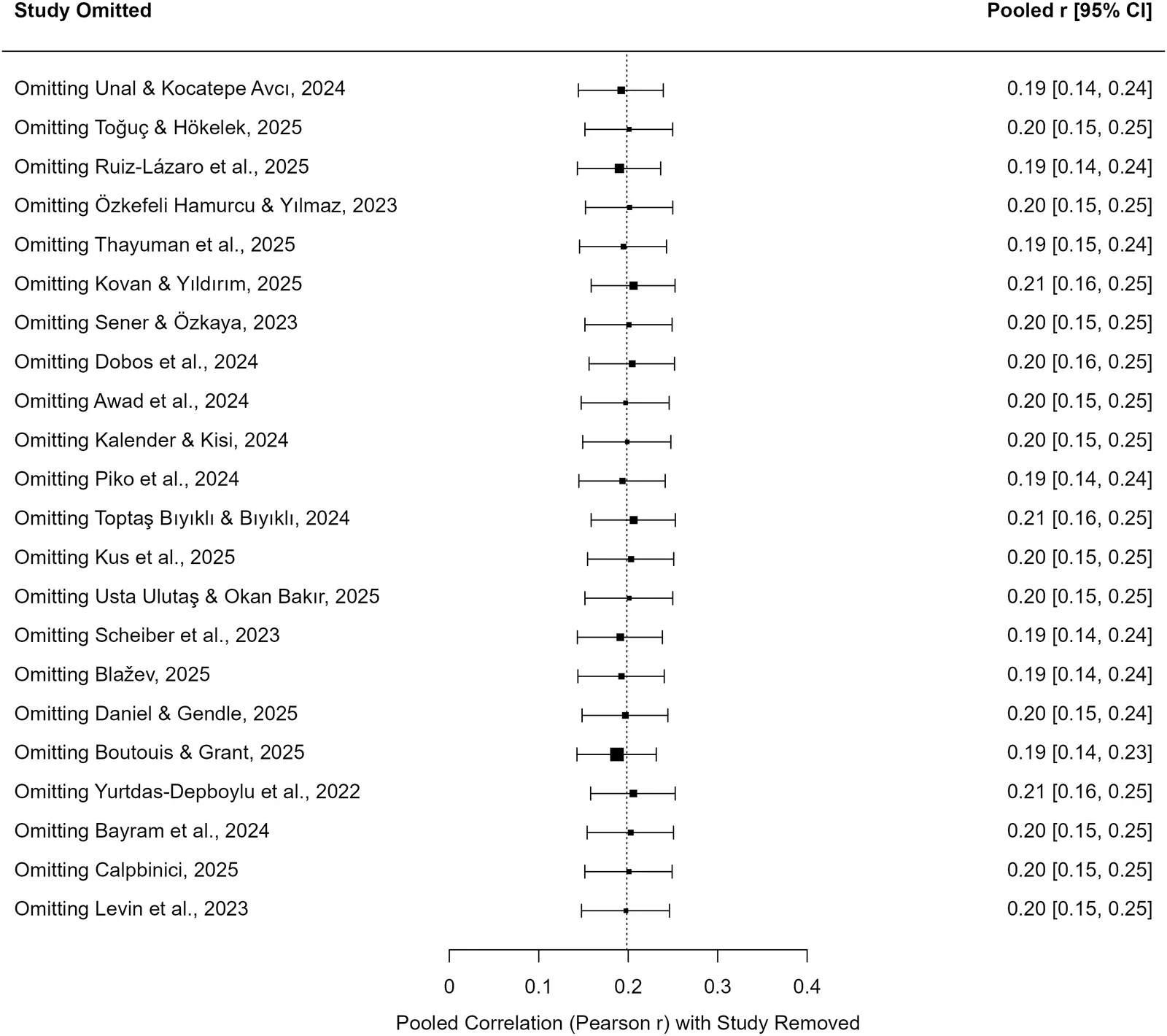
Leave-one-out sensitivity analysis of the pooled correlation.

**Fig 5:**
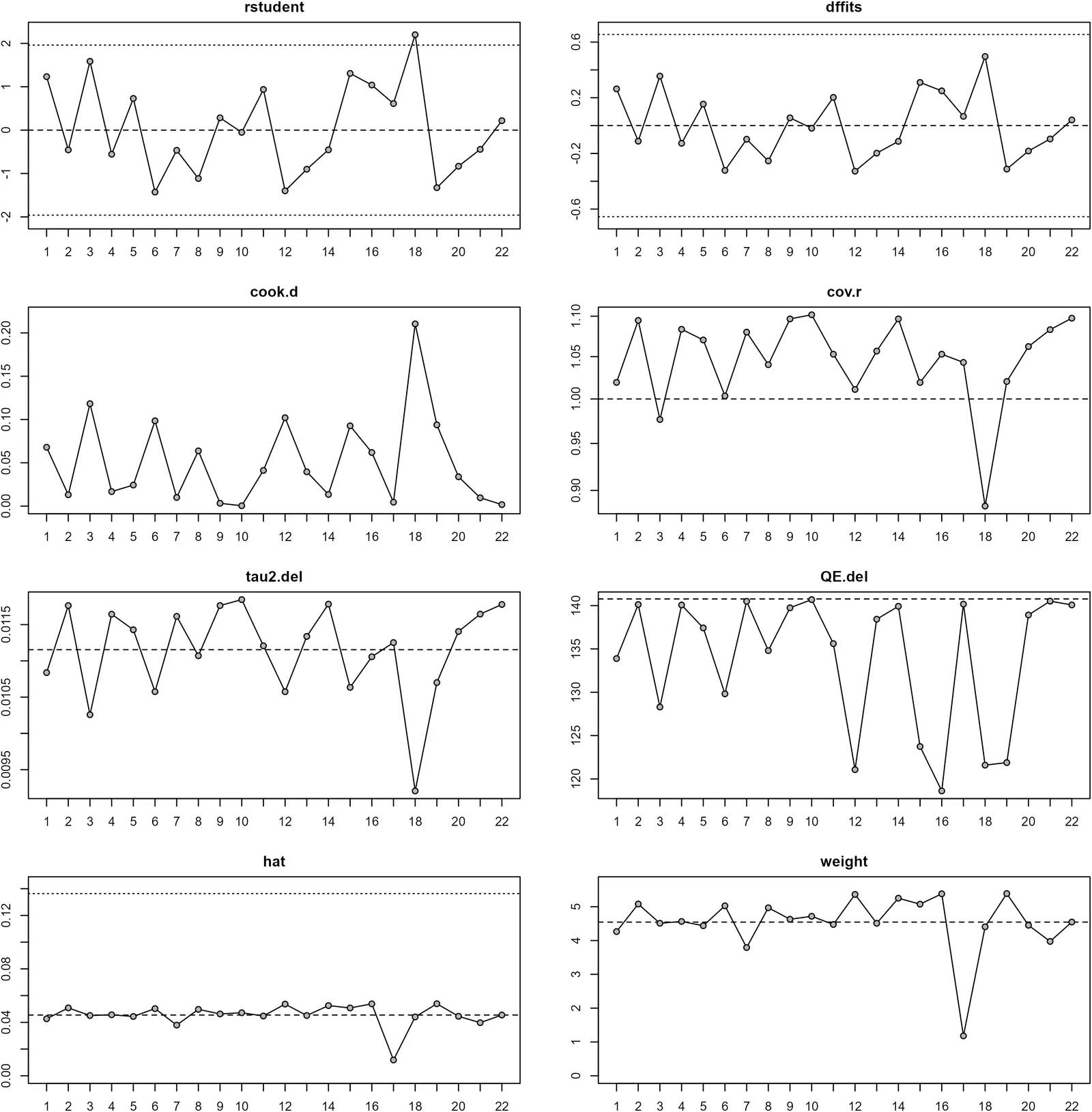
Influence diagnostics for the meta-analysis.

**Fig 6:**
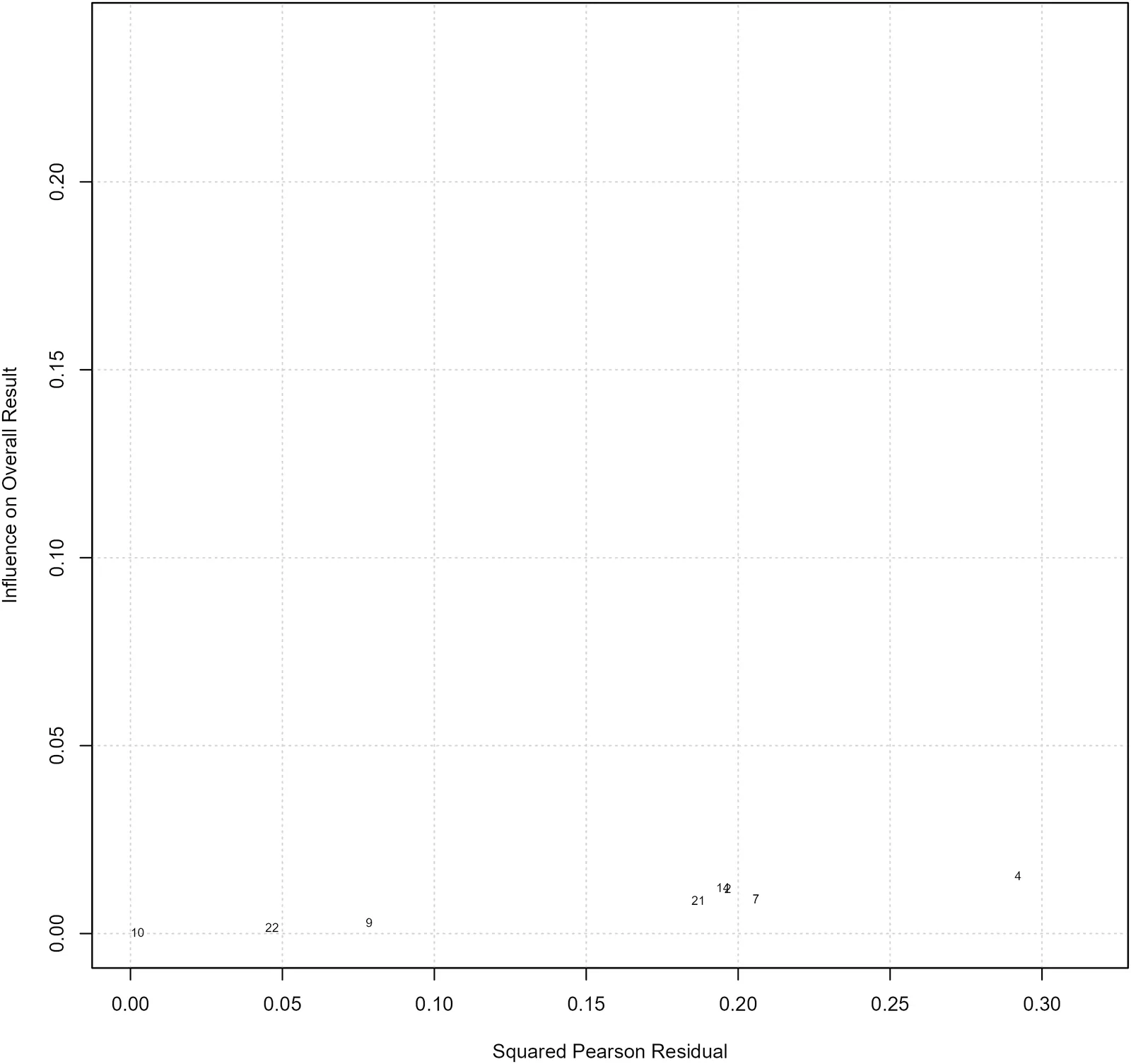
Baujat plot of study influence and contribution to heterogeneity.

**Fig 7:**
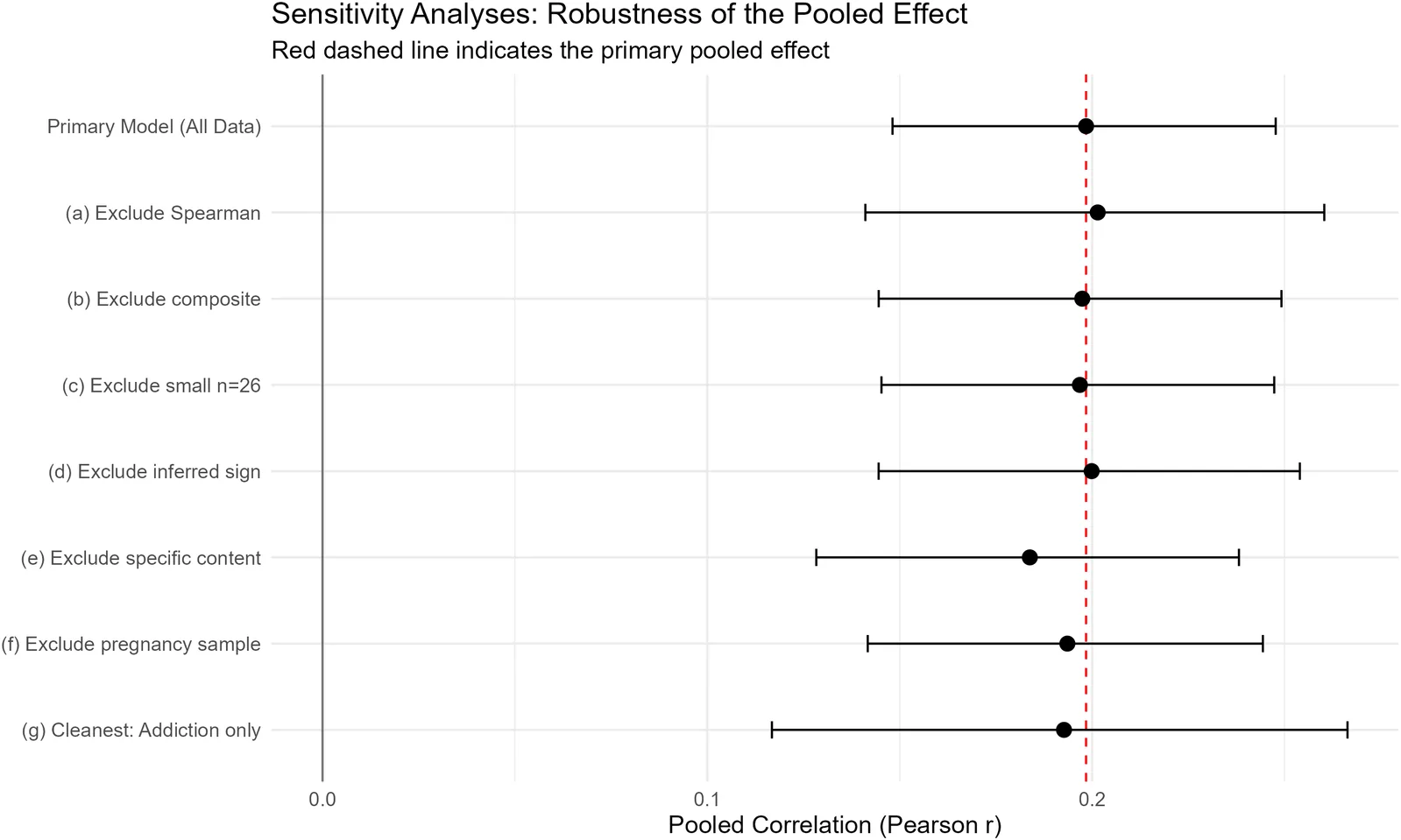
Sensitivity analyses of the pooled correlation.

**Publication bias.** A contour-enhanced funnel plot (Fig 8) showed studies falling predominantly within the higher-significance contours to the right of the null. The Duval–Tweedie trim-and-fill procedure imputed 4 hypothetical missing studies on the left side of the funnel, yielding a bias-adjusted pooled correlation of r = 0.17 (95% CI, 0.12 to 0.22). This downward adjustment is modest, and the association remained positive and statistically significant after correction, indicating that while the funnel shows mild asymmetry, plausible publication bias is unlikely to overturn the primary finding. These procedures should be read cautiously, since trim-and-fill performs poorly when between-study heterogeneity is as high as that observed here, and with 22 studies tests for funnel asymmetry are of limited power. Meta-regression of effect size on log sample size showed only a non-significant tendency for smaller studies to report larger effects (b = −0.057, p = 0.14), which is consistent with mild asymmetry that is unlikely to overturn the finding.

**Fig 8:**
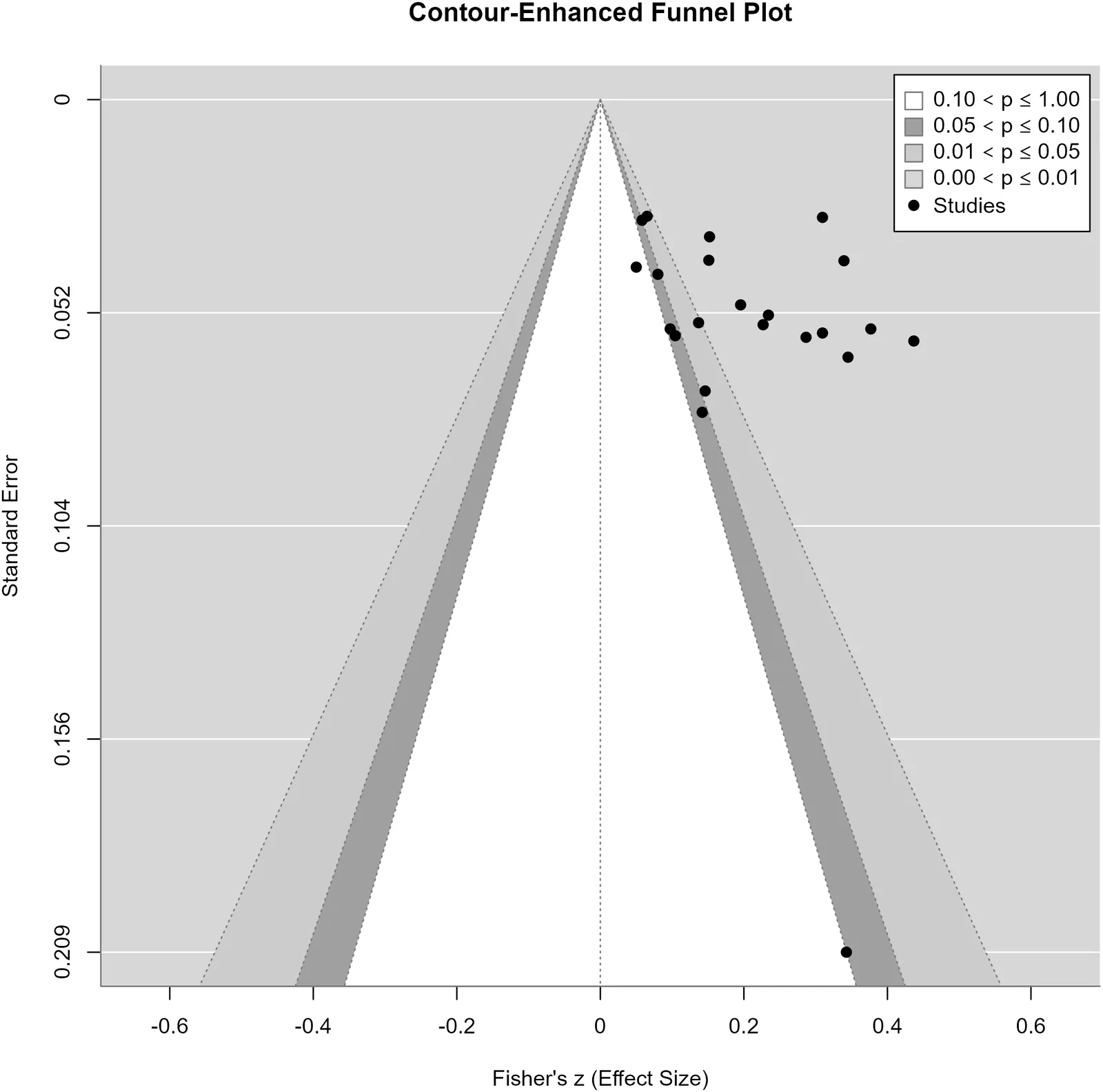
Contour-enhanced funnel plot assessing potential publication bias.

**Secondary dichotomous analysis.** Two studies providing reconstructable 2 × 2 cell counts, Tarsitano et al. 2022 (more than 60 versus fewer than 15 minutes per day; 525 of 1,693 versus 48 of 220) and Yilmazel 2021 (social media addiction score 74 or above versus 41–73; 492 of 764 versus 111 of 205), both back-calculated from published band prevalences and addiction-score categories rather than reported directly, were pooled in a dichotomous comparison of orthorexia by level of social media use (Fig 9). Individuals with higher social media use had significantly greater odds of orthorexia (pooled OR = 1.57, 95% CI 1.25 to 1.97), with no detectable heterogeneity (I² = 0%, p = .83).

**Fig 9:**
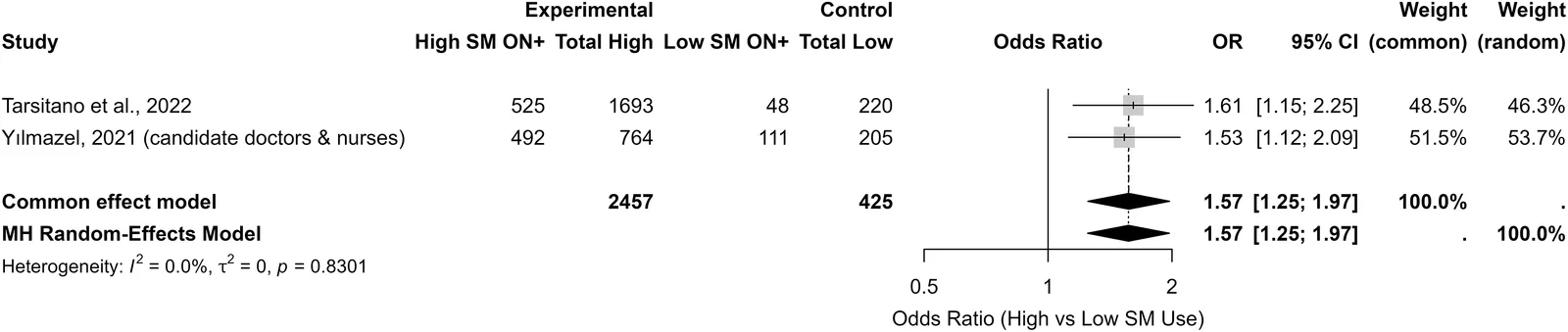
Dichotomous comparison of orthorexia nervosa according to level of social media use.

**Pooled prevalence.** Descriptive pooled prevalence estimates, stratified by instrument and cutoff, showed substantial variability. Pooled prevalence was estimated only within single-cutoff strata. Among studies using the conservative ORTO-15 cutoff below 35 it was 39.9% (95% CI 30.8 to 49.4; I² = 93.1%; Fig 10), and among studies using the over-inclusive cutoff below 40 it rose to 65.5% (95% CI 48.7 to 80.5; I² = 98.7%; Fig 11). Prevalence reported with the ORTO-11 was not pooled, because the five contributing studies applied at least three different cutoffs, two of which could not be confirmed from the source reports; observed prevalence across them ranged from 23.0% to 67.7% (Fig 12). Given the extreme heterogeneity across all three pooled prevalence estimates, these figures should be interpreted as descriptive summaries of highly variable underlying rates rather than precise population estimates.

**Fig 10:**
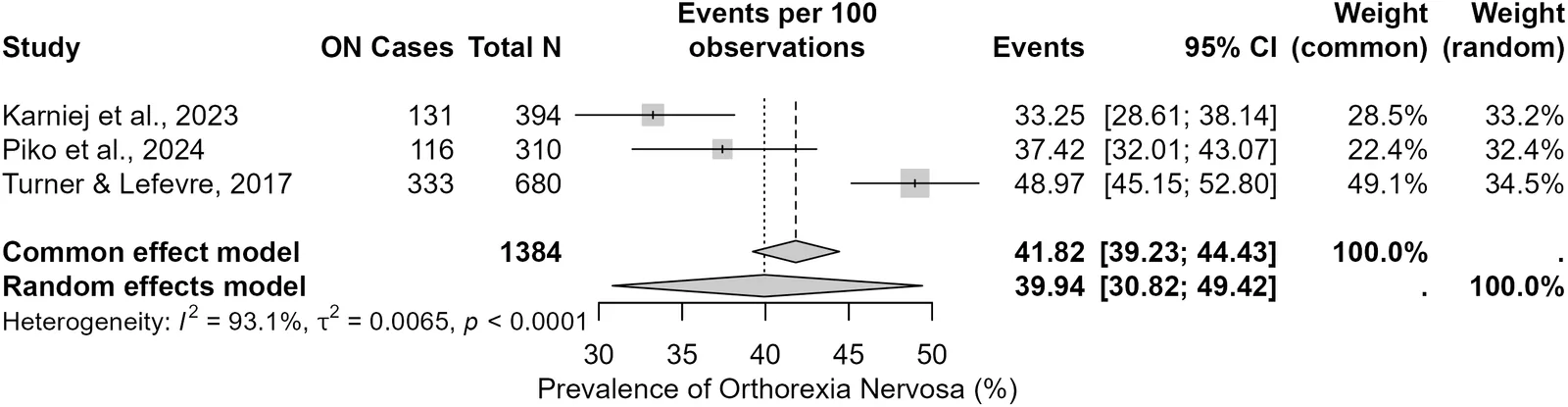
Pooled prevalence of ON using the ORTO-15 cutoff below 35.

**Fig 11:**
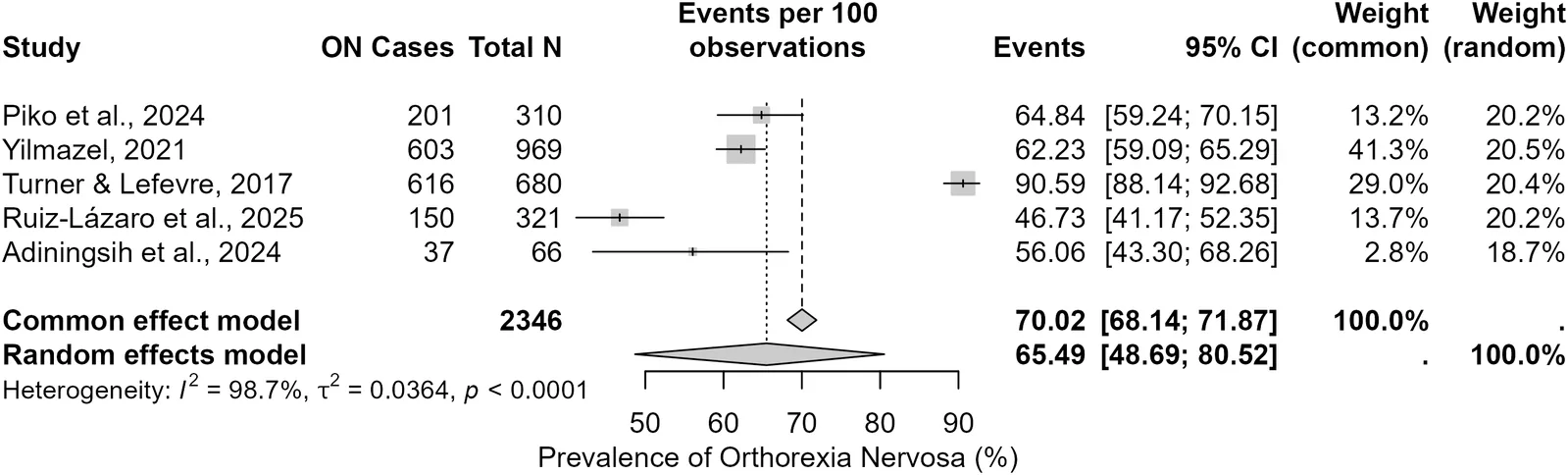
Pooled prevalence of ON using ORTO-15 cutoff below 40.

**Fig 12:**
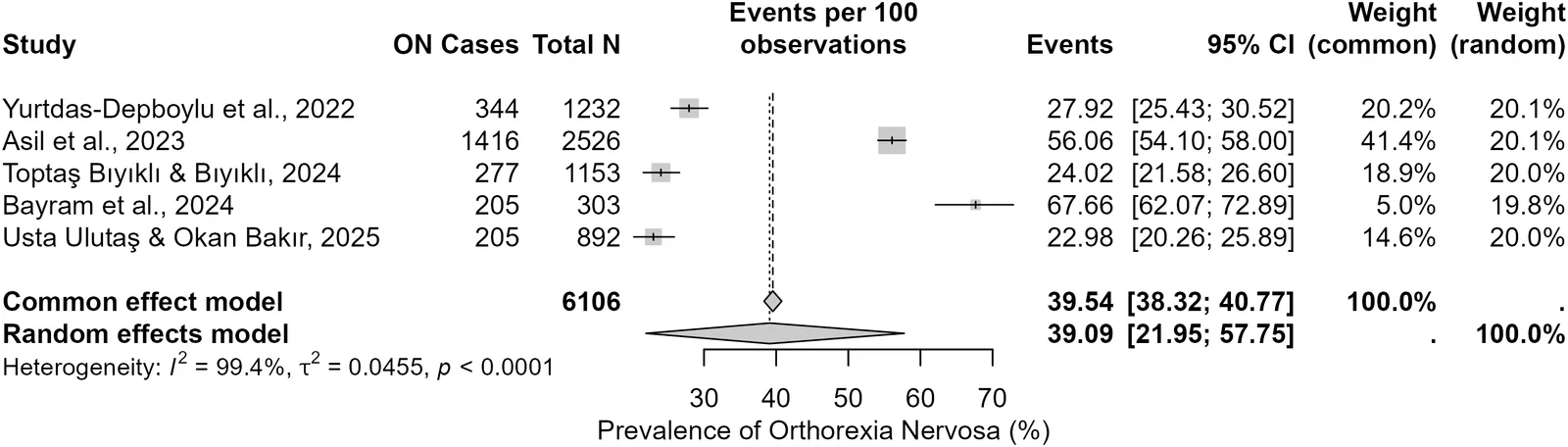
Observed prevalence of ON using the ORTO-11.

**Certainty of evidence.** Certainty in the primary pooled correlation was assessed using GRADE. Starting from a low baseline (all contributing studies were observational and cross-sectional), the evidence was downgraded once for serious inconsistency (I² = 84%, with the scale-scoring-direction subgroup accounting for roughly a quarter of this heterogeneity) and and was not downgraded for imprecision, since the confidence interval around the pooled estimate (0.15 to 0.25) excludes the null and is narrow and the accrued sample of 10,868 participants exceeds any plausible optimal information size; the width of the prediction interval reflects between-study heterogeneity and is accounted for under inconsistency rather than counted a second time. Certainty was not downgraded for indirectness, as all studies measured social media exposure and orthorexia symptoms directly in the populations of interest, nor for risk of bias, as excluding the single study at high risk of bias left the pooled estimate unchanged, nor for publication bias, as the trim-and-fill adjusted estimate (r = 0.17, 95% CI 0.12 to 0.22) remained positive and statistically significant, yielding an overall certainty of **very low**. In summary, there is very low-certainty evidence of a small, positive cross-sectional association (r ≈ 0.20) between social media use and orthorexia nervosa; because all contributing evidence is cross-sectional, this association cannot establish that social media use increases orthorexia over time.

## Discussion

This systematic review and meta-analysis included 33 studies examining the association between social media use and ON, 22 of which contributed to a pooled correlational analysis. Across those 22 studies the pooled correlation was small but consistent in direction (r = 0.20, 95% CI 0.15 to 0.25), corresponding to roughly 4% of shared variance, and every harmonized coefficient was positive. Heterogeneity was substantial (I² = 84.3%) and the 95% prediction interval (−0.02 to 0.40) extends below zero, so a negligible association in some populations remains compatible with these data. About a quarter of that heterogeneity (R² = 26.7%) was attributable to whether the orthorexia instrument was reverse-scored, which supports the concern that ORTO-family measures attenuate the association by capturing normative healthy eating alongside pathology. Certainty in the pooled estimate was very low by GRADE, so the finding is best described as promising rather than established. Most studies demonstrated a positive association suggesting that social media use is associated wtih orthorexic tendencies. The type of content an individual is exposed to was more consistently associated with orthorexic tendencies than platform or duration of use, with health and food related content being most associated. Additionally, psychological factors were found to have an important role with body dissatisfaction, perfectionism and emotion regulation playing a pivotal role in this association.

The measurement of orthorexia is the most serious limitation of this evidence base, and it constrains how our pooled estimate should be read. The prominent and significant limitations in the existing literature on ON and orthorexic tendencies, especially with respect to the measurement tools. Twenty-four of the 33 included studies (and 14 of the 22 contributing to the pooled correlation) used the ORTO-15, the ORTO-11 or the ORTO-R. This instrument family has well-documented psychometric limitations across multiple validation studies, has been shown to have considerable psychometric limitations across multiple validation studies [70,71]. The ORTO-15 was shown to overpathologize healthy eating habits, not adequately distinguishing between truly pathological orthorexic behaviors and non-pathological interest in healthy eating [71,72]. Additionally, inconsistent factorial structures, ambiguous item wording, poor internal consistency, and inflated prevalence rates were noted when utilizing this tool [71,72]. Taken together, the evidence indicates that the ORTO instruments are sensitive to dietary restriction but do not discriminate the pathological form of orthorexia, and should be regarded as screening rather than diagnostic tools.

Our own analyses quantify how much this matters. First, scoring direction was the only moderator to explain a significant share of the between-study variance: the pooled correlation was 0.26 (95% CI 0.19 to 0.33) among the 10 studies using normally scored instruments but 0.15 (95% CI 0.08 to 0.21) among the 12 using reverse-scored ORTO measures, accounting for 26.7% of the heterogeneity. Second, the instrument family itself did not explain a significant share of the variance (F(4,17) = 0.35, p = 0.84), with the ORTO family yielding 0.18 (95% CI 0.11 to 0.24) against 0.22 to 0.24 for the DOS, TOS, ONI and EHQ, so the attenuation tracks how an instrument is scored rather than which instrument it is. Third, and most starkly, the estimated prevalence of orthorexia in studies using the same instrument shifted from 39.9% at an ORTO-15 cutoff below 35 to 65.5% at a cutoff below 40, and the five ORTO-11 studies could not be pooled at all because no two applied the same threshold. Measurement choice therefore changes the apparent burden of orthorexia more than any population characteristic we examined.

Accordingly, the pooled correlation reported here is likely to be a conservative estimate of the association, since the majority of contributing studies used the instrument family that attenuates it. Future primary studies should report results using an instrument with a stable factor structure, such as the DOS, TOS, ONI or EHQ, state the cutoff applied, and report prevalence at more than one threshold so that estimates remain comparable. Many of the studies heavily rely on ORTO-15 or its variations for assessment; thus, these psychometric issues should be considered when interpreting prevalence rates and associations reported in the ON literature where there might be overestimation of true ON cases [70,71]

The findings of our studies align with the extensive body of literature documenting the association between exposure to social media and other eating disorders. A meta-analysis conducted by Zhang et. al [72] confirms that social networking sites use is significantly related to disordered eating behaviors and attitudes, a finding that’s consistent with the findings from the included studies in this review. In fact, slim and slender figures predominate the media and are presented as ideals. This presentation, however unrealistic and most of the time unachievable, puts heavy psychological pressure on users who are constantly exposed [73].

Beyond the frequency or duration of use, the nature of the content an individual is being exposed to appears to play an important role [36]. This finding was evident in our included studies [29,31,48,63], where exposure to fitness and pro-anorexia content, reading nutrition related posts, viewing and sharing healthy eating content, were associated with greater body dissatisfaction and with higher orthorexia scores. While the widespread presence of thin is consistently associated with disordered eating habits, the dissemination of misinformation on social media platforms is another factor consistently associated with these outcomes. With the usage of social media becoming ubiquitous [74], it has also become one of the most important and fastest-growing source of information, particularly for health and nutrition related information, due to its accessibility and convenience [75]. And while these platforms can act as a mean for health education [76], the lack of content regulation and verification strategies allow the rapid spread of misinformation and disinformation, particularly on topics related to nutrition [77–80] A prominent example highlighting the potential harms associated with misinformation can have on consumers is that of miracle diets. These are diets that promise immediate health improvement and are claimed to be curative and preventive for many health issues and diseases despite no scientific evidence. These diets are associated with ON, with all articles tackling ON also referencing miracle diets [81]. Moreover, more than 45% of dietary advice shared on social media platforms has been found to be inaccurate [82], an alarmingly elevated percentage, particularly with evidence that individuals with ON had a higher tendency to adopt social media advice rather than suggestions from professionals [83]. The increasing number of social media influencers may compound this problem. They are often seen as authority figures which gives their misleading, unscientific content, more credibility [84,85]

However, despite the widespread sociocultural pressure for thinness and healthy eating throughout the media, the impact on individuals varies significantly. Indeed, all the factors discussed earlier have been associated with eating disorders, but not all individuals internalize these ideals, and even when internalized, a few develop an eating disorder [86], highlighting individual differences in vulnerability. A meta-analysis by Hausenblas et al. effectively found that while exposure to body ideals on social media platforms do increase body dissatisfaction, this effect is rather seen in individuals at increased risk for eating disorders such as those who exhibit disordered eating behaviors [87]. Sociocultural, psychological and biological factors are thought to interact, and individual differences in the internalization of thin ideals have been linked to each of them. Personality traits, which are partially genetically determined, have been associated with greater vulnerability [88]. Perfectionism has been associated with an over-evaluation of weight and shape [88]. Additionally, problematic social media use, has been associated with greater emotion dysregulation and maladaptive perfectionism, with a greater reported need for external validation, and with more frequent social comparison to unrealistic lifestyles represented by influencers [89]. Moreover, those with perfectionistic tendencies often emphasize achievement and may feel guilty if they consider they’re not eating healthy “enough” [90,91] This adaptive perfectionism and the setting of such high standards for eating only the best diets has been proposed as one pathway to orthorexic tendencies, although no included study tested this sequence longitudinally [92,93]

Individuals at risk of developing eating disorders display cognitive biases that influence their processing of information and response to it, particularly information related to body image, weight and dieting. The concept of attentional bias [94] describes how such individuals tend to selectively focus on content related to food and body weight in addition to assigning increased importance to these contents even when exposed to other information [95,96], without evaluating their credibility and relevance [97–100]. This attention bias could help explain why diet misinformation particularly affect them more than others, based on their own fixation on such topics. Beyond attentional bias, susceptible individuals also show rigid adherence to their beliefs and reject information that contradict their views, regardless of their credibility [101–103] It’s worth noting that modern social media platforms, like Instagram, reinforce this vulnerability through their algorithmic promotion of selectively followed accounts [14]. Together these factors have been used to propose a pathway in which following accounts that promote healthy eating and exercise, undertaken with the expectation of improving health, is accompanied by a gradual shift toward more restrictive eating attitudes and, in some individuals, orthorexic tendencies. This pathway is consistent with the cross-sectional associations reported here but has not been demonstrated prospectively.

To sum it all up, several mechanisms play a role in the complex association between social media use and increased orthorexic tendencies. The dissemination of misinformation on social media platforms, especially by influencers seen as credible, endorses disordered eating behaviors by portraying them as healthy, “magic diets”. Additionally, individuals susceptible to eating disorders (due to sociocultural, psychological and genetic factors) exhibit attention biases and rigidity in their adherence to misinformation, and may be more responsive to suchharmful content, which may help explain why heavier engagement with this content is associated with higher orthorexia scores, although the direction of this relationship remains uncertain..

### Strengths and limitations

To our knowledge this is the first review to pool the association between social media use and orthorexia nervosa quantitatively. The mixed-methods scoping review by Awad et al. mapped this literature narratively and judged statistical pooling unsupportable; we restricted inclusion to quantitative studies using validated ON measures, appraised them with the Newcastle-Ottawa Scale, extended the search to January 2026, and report a pooled effect with pre-specified subgroup, sensitivity and small-study analyses and a GRADE certainty rating.It sheds light on an emerging and understudied, clinically relevant topic. Furthermore, the review contributes to public health awareness by highlighting potential risks of social media use, which could inform future regulations and targeted interventions.

Several limitations should be considered when interpreting these findings. First, all 33 included studies employed a cross-sectional design; consequently, no causal or temporal inference can be drawn from this evidence base. In particular, reverse causality — whereby individuals with pre-existing orthorexic tendencies selectively seek out and engage with health- and fitness-related social media content — remains an equally plausible explanation for the observed associations and cannot be ruled out with the available data. Second, the majority of included studies relied on the ORTO-11 or ORTO-15 instruments to assess ON, both of which have well-documented psychometric limitations, including unstable factor structure, inconsistent cutoff scores across cultural adaptations, and a tendency to over-pathologize normative healthy eating behavior. This measurement heterogeneity likely contributes to the substantial statistical heterogeneity observed in our pooled analysis (I² ≈ 84%) and limits the direct comparability of prevalence and correlation estimates across studies using different ON instruments. Relatedly, no standardized operationalization of the exposure (social media use) exists across the literature: included studies variously measured duration, frequency, addiction/problematic-use scores, platform type, and content type as proxies for “social media use,” which further limits comparability between studies and should be considered an underlying limitation of the evidence base rather than of the present synthesis alone. Third, only 22 of the 33 included studies (66.7%) reported a correlational effect size that could be pooled quantitatively; the remaining studies used odds ratios, group comparisons, or qualitative designs that precluded statistical pooling. The pooled correlation reported here (r = 0.20, 95% CI 0.15 to 0.25) should therefore not be interpreted as representative of the full body of included evidence, but rather as a synthesis of the subset of studies reporting a comparable effect size. Overall certainty in this pooled estimate was rated very low using the GRADE framework, reflecting concerns about inconsistency, risk of bias, and imprecision, and readers should weigh the pooled effect accordingly. Fourth, included studies varied considerably in sample size (ranging from 27 to 4,107 participants, and from 26 to 1,232 within the meta-analyzed subset). While the meta-analyzed subset was formally weighted by inverse variance, studies retained in the narrative synthesis were not similarly weighted, meaning findings from smaller studies in that track should be interpreted with additional caution. Similarly, although the single study rated at high risk of bias was excluded in a sensitivity analysis of the meta-analyzed subset without material change to the pooled estimate, these same studies remain part of the narrative synthesis, where their contribution could not be adjusted for in the same way. Finally, contradictory findings regarding gender and body mass index across included studies could not be formally reconciled through meta-regression or subgroup analysis, as too few studies reported these variables with extractable, comparable effect sizes; these inconsistencies are therefore discussed narratively rather than statistically resolved. Future research should prioritize longitudinal and experimental designs capable of studying causality and temporal relationship. The development and validation of psychometrically robust tools to assess ON are also needed to ensure precision in outcome assessment. Furthermore, future studies should explore protective factors and effective interventions to mitigate the potential impact of social media on disordered eating behaviors in general, and ON in particular.

## Conclusion

This systematic review highlights a growing and consistent body of evidence linking orthorexic tendencies with social media use, particularly in relation to fitness, diet, and health-related content. The majority of included observational studies found that higher levels of social media use — particularly on Instagram and TikTok — were associated with greater orthorexic tendencies, alongside co-occurring factors such as body image dissatisfaction, emotion dysregulation, and perfectionism. The type of content consumed online emerged as a consistent correlate of orthorexic tendencies, appearing more informative than platform or duration alone. Repeated exposure to idealized body and health imagery was consistently associated with higher ON scores across studies, although, given the cross-sectional design of all included studies, this evidence cannot establish whether such exposure precedes or follows the development of orthorexic tendencies; it remains equally plausible that individuals with pre-existing orthorexic tendencies selectively seek out this type of content.

Taken together, these findings suggest that social media use may represent a relevant modern contextual factor associated with eating-disorder symptoms and obsessive behaviors such as ON, particularly among adolescents and young adults who may be more susceptible to unrealistic body ideals and health-related misinformation. While causality cannot be inferred from the current evidence, these associations nonetheless point to the potential value of media literacy interventions, platform-level content moderation, and public health awareness efforts as precautionary measures, pending confirmation through more rigorous study designs.

Future research should prioritize longitudinal and experimental designs to clarify the direction of these associations and to test the effectiveness of strategies aimed at reducing potentially harmful patterns of social media engagement. Proactive, evidence-informed approaches remain warranted to support vulnerable populations in navigating misleading or unregulated health-related content online, with the goal of fostering safer and more informed social media use.

## Supporting information

S1 Text Complete search strategies for all databases. (DOCX)

S1 Data Full-text screening of potentially eligible studies. (XLSX)

S1 Table Newcastle-Ottawa Scale risk-of-bias assessment: individual domain and total scores for every included study. (DOCX)

S1 PRISMA Checklist PRISMA 2020 checklist. (DOCX)

## References

[1] Saraswati P, Nagendraswamy C. Orthorexia nervosa- A review. Int J Psychiatr Nurs. 2015;1(2):2. doi: 10.5958/2395-180X.2015.00062.6

[2] Chenkov Y, Hristova DN. A brief literature overview on orthorexia nervosa – one new representative in cluster of eating disorders. JofIMAB. 2021;27(1):3568–71. doi: 10.5272/jimab.2021271.3568

[3] Bratman S. Orthorexia vs. theories of healthy eating. Eat Weight Disord. 2017;22(3):381–5. doi: 10.1007/s40519-017-0417-6 28741285

[4] Brytek-Matera A. Orthorexia nervosa – An eating disorder, obsessive-compulsive disorder or disturbed eating habit?. Arch Psychiatry Psychother. 2023.

[5] Donini LM, Barrada JR, Barthels F, Dunn TM, Babeau C, Brytek-Matera A, et al. A consensus document on definition and diagnostic criteria for orthorexia nervosa. Eat Weight Disord. 2022;27(8):3695–711. doi: 10.1007/s40519-022-01512-5 36436144 PMC9803763

[6] Horovitz O, Argyrides M. Orthorexia and orthorexia nervosa: A comprehensive examination of prevalence, risk factors, diagnosis, and treatment. Nutrients. 2023;15(17):3851. doi: 10.3390/nu15173851 37686883 PMC10490497

[7] Donini LM, Marsili D, Graziani MP, Imbriale M, Cannella C. Orthorexia nervosa: A preliminary study with a proposal for diagnosis and an attempt to measure the dimension of the phenomenon. Eat Weight Disord. 2004;9(2):151–7. doi: 10.1007/BF03325060 15330084

[8] Łucka I, Janikowska-Hołoweńko D, Domarecki P, Plenikowska-Ślusarz T, Domarecka M. Orthorexia nervosa - A separate clinical entity, a part of eating disorder spectrum or another manifestation of obsessive-compulsive disorder?. Psychiatr Pol. 2019;53(2):371–82. doi: 10.12740/PP/OnlineFirst/85729 31317964

[9] Cena H, Barthels F, Cuzzolaro M, Bratman S, Brytek-Matera A, Dunn T, et al. Definition and diagnostic criteria for orthorexia nervosa: A narrative review of the literature. Eat Weight Disord. 2019;24(2):209–46. doi: 10.1007/s40519-018-0606-y 30414078

[10] Smailhodzic E, Hooijsma W, Boonstra A, Langley DJ. Social media use in healthcare: A systematic review of effects on patients and on their relationship with healthcare professionals. BMC Health Serv Res. 2016;16(1):442. doi: 10.1186/s12913-016-1691-0 27562728 PMC5000484

[11] Pellegrino A, Stasi A, Bhatiasevi V. Research trends in social media addiction and problematic social media use: A bibliometric analysis. Front Psychiatry. 2022;13:1017506. doi: 10.3389/fpsyt.2022.1017506 36458122 PMC9707397

[12] Perski O, Blandford A, Ubhi HK, West R, Michie S. Smokers’ and drinkers’ choice of smartphone applications and expectations of engagement: A think aloud and interview study. BMC Med Inform Decis Mak. 2017;17(1):25. doi: 10.1186/s12911-017-0422-8 28241759 PMC5329928

[13] Bányai F, Zsila Á, Király O, Maraz A, Elekes Z, Griffiths MD, et al. Problematic social media use: Results from a large-scale nationally representative adolescent sample. PLoS One. 2017;12(1):e0169839. doi: 10.1371/journal.pone.0169839 28068404 PMC5222338

[14] Turner PG, Lefevre CE. Instagram use is linked to increased symptoms of orthorexia nervosa. Eat Weight Disord. 2017;22(2):277–84. doi: 10.1007/s40519-017-0364-2 28251592 PMC5440477

[15] Syurina EV, Bood ZM, Ryman FVM, Muftugil-Yalcin S. Cultural phenomena believed to be associated with orthorexia nervosa - Opinion study in dutch health professionals. Front Psychol. 2018;9:1419. doi: 10.3389/fpsyg.2018.01419 30271356 PMC6142186

[16] Rodgers RF, Melioli T. The relationship between body image concerns, eating disorders and internet use, part I: A review of empirical support. Adolescence Research Review. 2016;1(2):95–119. doi: 10.1007/s40894-015-0016-6

[17] Turner PG, Lefevre CE. Instagram use is linked to increased symptoms of orthorexia nervosa. Eat Weight Disord. 2017;22(2):277–84. doi: 10.1007/s40519-017-0364-2 28251592 PMC5440477

[18] Gkiouleka M, Stavraki C, Sergentanis TN, Vassilakou T. Orthorexia nervosa in adolescents and young adults: A literature review. Children. 2022;9(3):365. doi: 10.3390/children9030365 PMC8947656 35327737

[19] Saul J, Rodgers RF, Saul M. Adolescent eating disorder risk and the social online world: An update. Child Adolesc Psychiatr Clin N Am. 2022;31(1):167–77. doi: 10.1016/j.chc.2021.09.004 34801153

[20] Saul JS, Rodgers RF. Adolescent eating disorder risk and the online world. Child Adolesc Psychiatr Clin N Am. 2018;27(2):221–8. doi: 10.1016/j.chc.2017.11.011 29502748

[21] Campaioli G, Sale E, Simonelli A, Pomini V. The dual value of the web: Risks and benefits of the use of the internet in disorders with a self-destructive component in adolescents and young adults. Contemp Fam Ther. 2017;39(4):301–13. doi: 10.1007/s10591-017-9443-9

[22] Thompson JK, Heinberg LJ, Altabe M, Tantleff-Dunn S. Sociocultural theory: The media and society. Exacting Beauty: Theory, Assessment, and Treatment of Body Image Disturbance. American Psychological Association. 1999. p. 85–124. doi: 10.1037/10312-003

[23] Stice E. Review of the evidence for a sociocultural model of bulimia nervosa and an exploration of the mechanisms of action. Clin Psychol Rev. 1994;14(7):7. doi: 10.1016/0272-7358(94)90002-7

[24] Tiggemann M. The role of media exposure in adolescent girls’ body dissatisfaction and drive for thinness: Prospective results. J Soc Clin Psychol. 2006;25(5):5. doi: 10.1521/jscp.2006.25.5.523

[25] Myers TA, Crowther JH. Social comparison as a predictor of body dissatisfaction: A meta-analytic review. J Abnorm Psychol. 2009;118(4):683–98. doi: 10.1037/a0016763 19899839

[26] Krawczyk R, Thompson JK. The effects of advertisements that sexually objectify women on state body dissatisfaction and judgments of women: The moderating roles of gender and internalization. Body Image. 2015;15:109–19. doi: 10.1016/j.bodyim.2015.08.001 26363356

[27] Harper B, Tiggemann M. The Effect of thin ideal media images on Women’s self-objectification, mood, and body image. Sex Roles. 2007;58(9–10):649–57. doi: 10.1007/s11199-007-9379-x

[28] Tiggemann M, Miller J. The Internet and adolescent girls’ weight satisfaction and drive for thinness. Sex Roles. 2010;63(1–2):1–2. doi: 10.1007/s11199-010-9789-z

[29] Silva SC, Elmashhara MG, Sousa MI. The body dissatisfaction role in the adoption of compulsive healthy eating behaviors. Int Rev Public Nonprofit Mark. 2022;20(4):853–73. doi: 10.1007/s12208-022-00357-z

[30] Scheiber R, Diehl S, Karmasin M. Socio-cultural power of social media on orthorexia nervosa: An empirical investigation on the mediating role of thin-ideal and muscular internalization, appearance comparison, and body dissatisfaction. Appetite. 2023;185:106522. doi: 10.1016/j.appet.2023.106522 36893917

[31] Blackburn MR, Hogg RC. #ForYou? the impact of pro-ana TikTok content on body image dissatisfaction and internalisation of societal beauty standards. PLoS One. 2024;19(8):e0307597. doi: 10.1371/journal.pone.0307597 39110711 PMC11305550

[32] Tiggemann M. Television and adolescent body image: The role of program content and viewing motivation. J Soc Clin Psychol. 2005;24(3):3. doi: 10.1521/jscp.24.3.361.65623

[33] Grabe S, Hyde JS, Lindberg SM. Body objectification and depression in adolescents: The role of gender, shame, and rumination. Psychological Women Quarterly. 2007;31(2):2. doi: 10.1111/j.1471-6402.2007.00350.x

[34] Johnson F, Wardle J. Dietary restraint, body dissatisfaction, and psychological distress: A prospective analysis. J Abnorm Psychol. 2005;114(1):1. doi: 10.1037/0021-843X.114.1.119 15709818

[35] Neumark-Sztainer D, Paxton SJ, Hannan PJ, Haines J, Story M. Does body satisfaction matter? Five-year longitudinal associations between body satisfaction and health behaviors in adolescent females and males. J Adolesc Health. 2006;39(2):2. doi: 10.1016/j.jadohealth.2005.12.001 16857537

[36] Sanzari CM, Gorrell S, Anderson LM, Reilly EE, Niemiec MA, Orloff NC, et al. The impact of social media use on body image and disordered eating behaviors: Content matters more than duration of exposure. Eat Behav. 2023;49:101722. doi: 10.1016/j.eatbeh.2023.101722 37060807 PMC10363994

[37] Awad E, Alleva JM, El Khoury C, Chamma N, Martijn C, Rizk R. Orthorexia nervosa and social media: A mixed-methods scoping review using a systematic methodology. PLoS One. 2026;21(3):e0340219. doi: 10.1371/journal.pone.0340219 41811871 PMC12978498

[38] Page MJ, McKenzie JE, Bossuyt PM, Boutron I, Hoffmann TC, Mulrow CD, et al. The PRISMA 2020 statement: An updated guideline for reporting systematic reviews. BMJ. 2021;372:n71. doi: 10.1136/bmj.n71 33782057 PMC8005924

[39] Newcastle—Ottawa Quality Assessment Scale (adapted for cross sectional studies). doi: 10.1371/journal.pone.0147601.s001

[40] Dobos B, Berki T, Mellor D, Piko BF. Mindful eating and orthorexia nervosa: How do they interact?. Nutr Bull. 2024;49(4):513–25. doi: 10.1111/nbu.12708 39262058

[41] Daniel G, Gendle MH. Relationships between orthorexia, exercise dependency, body image, and decision-making in university undergraduates. 2023.

[42] Kalender S, Kisi M. A study on health nutrition obsession, social-physique anxiety, and social media addiction of young individuals. Addicta- Turk J Addict. 2024;11(1). doi: 10.5152/ADDICTA.2024.23141

[43] Staśkiewicz-Bartecka W, Kalpana K, Aktaş S, Khanna GL, Zydek G, Kardas M, et al. The impact of social media and socio-cultural attitudes toward body image on the risk of orthorexia among female football players of different nationalities. Nutrients. 2024;16(18):3199. doi: 10.3390/nu16183199 39339799 PMC11435300

[44] Unal GA, Kocatepe Avcı U. The mediating role of attitudes towards eating in the relationship between compulsive instagram use and orthorexia in adults. Psychol Res Behav Manag. 2024;17:4237–48. doi: 10.2147/PRBM.S491268 39679318 PMC11646376

[45] Şener BS, Özkaya H. Investigation of the relationship between social media addiction and orthorexia nervosa in adult individuals who applied to obesity polyclinic. Addicta: The Turkish Journal on Addictions. 2023;10(2):135–41. doi: 10.5152/addicta.2023.23042

[46] Asil E, Yılmaz MV, Ayyıldız F, Yalçın T. The effect of social media use on orthorexia nervosa: A sample from Turkey. Nutr Hosp. 2023;40(2):384–90. doi: 10.20960/nh.04217 36880720

[47] Karniej P, Pérez J, Juárez-Vela R, Santolalla-Arnedo I, Gea-Caballero V, Del Pozo-Herce P, et al. Orthorexia nervosa in gay men-the result of a spanish-polish eating disorders study. BMC Public Health. 2023;23(1):58. doi: 10.1186/s12889-022-14943-7 36624429 PMC9830745

[48] Levin RL, Mills JS, McComb SE, Rawana JS. Examining orthorexia nervosa: Using latent profile analysis to explore potential diagnostic classification and subtypes in a non-clinical sample. Appetite. 2023;181:106398. doi: 10.1016/j.appet.2022.106398 36455786

[49] Gobin KC, Mills JS, McComb SE. The effects of the COVID-19 pandemic lockdown on eating, body image, and social media habits among women with and without symptoms of orthorexia nervosa. Front Psychol. 2021;12:716998. doi: 10.3389/fpsyg.2021.716998 34975611 PMC8714632

[50] Kuş Y, Tan E, Doğan O, Bas A. From likes to obsessions: The mediating role of obsessive-compulsive symptoms in the relationship between social media addiction and orthorexia nervosa. J Clin Psy. 2025;28(1):7–15. doi: 10.5505/kpd.2025.34979

[51] Toptaş Bıyıklı E, Bıyıklı AE. Evaluation of orthorexia nervosa tendency and social media addiction in university students. Genel Tıp Dergisi. 2024;34(6):867–74. doi: 10.54005/geneltip.1557960

[52] Christodoulou E, Markopoulou V, Koutelidakis AE. Exploring the link between mindful eating, instagram engagement, and eating disorders: A focus on orthorexia nervosa. Psychiatry International. 2024;5(1):27–38. doi: 10.3390/psychiatryint5010003

[53] Özkefeli Hamurcu TG, Yılmaz S. The correlation between orthorexia nervosa and social media use in nursing students. İnönü Üniversitesi Sağlık Hizmetleri Meslek Yüksek Okulu Dergisi. 2023;11(1):1144–58. doi: 10.33715/inonusaglik.1156789

[54] Kovan A, Yıldırım M. Social media use, eating attitudes, orthorexia nervosa and well-being: Testing a moderated mediation model. Eat Weight Disord. 2025;30(1):79. doi: 10.1007/s40519-025-01753-0 41037224 PMC12491097

[55] Ruiz-Lázaro J, Cuesta-Zamora C, Ros L. The role of social media addiction, depression, rumination and disordered eating behaviours in orthorexia nervosa symptoms. Psychol Rep. 2025;:332941251363905. doi: 10.1177/00332941251363905 40762697

[56] Thayuman J, Whitehead R, Borthwick C, Argent T, Williams J. Nonattachment to self mediates the relationship between Instagram engagement and orthorexia nervosa amongst Australian university students. Appetite. 2025;215:108230. doi: 10.1016/j.appet.2025.108230 40675456

[57] Boutouis S, Grant JE. Problematic usage of the internet is associated with orthorexia nervosa tendency in adults. CNS Spectr. 2025;30(1):e96. doi: 10.1017/S1092852925100771 41383009 PMC13064715

[58] Adiningsih S, Wilson W, Tejoyuwono AAT. The relationship of body mass index, diet, and eating pattern with orthorexia nervosa amongst medical students. IJPHS. 2024;13(1):268. doi: 10.11591/ijphs.v13i1.23513

[59] Awad E, Rogoza R, Gerges S, Obeid S, Hallit S. Association of social media use disorder and orthorexia nervosa among lebanese university students: The indirect effect of loneliness and factor structure of the social media use disorder short form and the jong-gierveld loneliness scales. Psychol Rep. 2024;127(3):1065–84. doi: 10.1177/00332941221132985 36245332

[60] Piko BF, Kulmán E, Mellor D. Orthorexic tendency in light of eating disorder attitudes, social media addiction and regular sporting among young hungarian women. Issues Ment Health Nurs. 2024;45(9):990–7. doi: 10.1080/01612840.2024.2379428 39121495

[61] Bayram HM, Barcin-Guzeldere HK, Ede-Cintesun E, Margot Çelik ZMÇ. Exploring the interplay between social media addiction, mindful eating, intuitive eating, orthorexia nervosa, and mental health in young adults. Nor Afr J Food Nutr Res. 2024;8(18):70–9. doi: 10.51745/najfnr.8.18.70-79

[62] Tarsitano MG, Pujia R, Ferro Y, Mocini E, Proni G, Lenzi FR, et al. Symptoms of Orthorexia Nervosa are associated with time spent on social media: A web-based survey in an Italian population sample. Eur Rev Med Pharmacol Sci. 2022;26(24):9327–35. doi: 10.26355/eurrev_202212_30683 36591841

[63] Yurtdas-Depboylu G, Kaner G, Özçakal S. The association between social media addiction and orthorexia nervosa, eating attitudes, and body image among adolescents. Eat Weight Disord-Stud Anorex Bulim Obes. 2022. doi: 10.1007/s40519-022-01521-4 36562891

[64] Yılmazel G. Orthorexia tendency and social media addiction among candidate doctors and nurses. Perspect Psychiatr Care. 2021;57(4):1846–52. doi: 10.1111/ppc.12758 33861475

[65] Blažev D. Biological, behavioural, psychological and social predictors of orthorexia nervosa among young women in Croatia. Drustvena Istraz. 2025;34(1):87–110. doi: 10.5559/di.34.1.05

[66] Usta Ulutaş P, Okan Bakır B. The type of social media is a greater influential factor for orthorexia nervosa than the duration: A cross-sectional study. BMC Public Health. 2025;25(1):827. doi: 10.1186/s12889-025-21335-0 40025461 PMC11871614

[67] Toğuç H, Hökelek B. Psychosocial effects of social media and nutrition information: An analysis of orthorexia nervosa and weight-related self-stigma in youth. BMC Psychol. 2025;13(1):1063. doi: 10.1186/s40359-025-03409-1 41013648 PMC12465448

[68] Calpbinici P. Effect of social media use on the orthorexia nervosa tendency among pregnant women. Pak J Med Sci. 2025;41(6):1755–62. doi: 10.12669/pjms.41.6.12008 40621537 PMC12223734

[69] Gobin KC, Mills JS, McComb SE. The effects of the COVID-19 pandemic lockdown on eating, body image, and social media habits among women with and without symptoms of orthorexia nervosa. Front Psychol. 2021;12:716998. doi: 10.3389/fpsyg.2021.716998 34975611 PMC8714632

[70] Donofre GS, Japur CC, Laus MF, Alvarenga MDS, Silva WRD. Psychometric properties of various factorial models of the ORTO-15 tested with Brazilian respondents. Percept Mot Skills. 2024;131(5):1531–47. doi: 10.1177/00315125241267134 39033355

[71] Roncero M, Barrada JR, Perpiñá C. Measuring orthorexia nervosa: Psychometric limitations of the ORTO-15. Span J Psychol. 2017;20:E41. doi: 10.1017/sjp.2017.36 28929989

[72] Zhang J, Wang Y, Li Q, Wu C. The relationship between SNS usage and disordered eating behaviors: A meta-analysis. Front Psychol. 2021;12:641919. doi: 10.3389/fpsyg.2021.641919 34413807 PMC8367749

[73] Carrotte ER, Prichard I, Lim MSC. “Fitspiration” on social media: A content analysis of gendered images. J Med Internet Res. 2017;19(3):e95. doi: 10.2196/jmir.6368 28356239 PMC5390113

[74] Social Media Fact Sheet. Pew Research Center. 2024. Accessed April 17, 2025. https://www.pewresearch.org/internet/fact-sheet/social-media/

[75] Suarez-Lledo V, Alvarez-Galvez J. Prevalence of health misinformation on social media: Systematic review. J Med Internet Res. 2021;23(1):e17187. doi: 10.2196/17187 PMC7857950 33470931

[76] Saha K, Torous J, Ernala SK, Rizuto C, Stafford A, De Choudhury M. A computational study of mental health awareness campaigns on social media. Transl Behav Med. 2019;9(6):1197–207. doi: 10.1093/tbm/ibz028 30834942 PMC6875652

[77] Pagoto S, Waring ME, Xu R. A call for a public health agenda for social media research. J Med Internet Res. 2019;21(12):e16661. doi: 10.2196/16661 31855185 PMC6940852

[78] Kouzy R, Abi Jaoude J, Kraitem A. Coronavirus goes viral: Quantifying the COVID-19 misinformation epidemic on Twitter. Cureus. 2020. doi: 10.7759/cureus.7255 PMC7152572 32292669

[79] Fiuza A, Rodgers RF. The effects of brief diet and anti-diet social media videos on body image and eating concerns among young women. Eat Behav. 2023;51:101811. doi: 10.1016/j.eatbeh.2023.101811 37708720

[80] Hruska J, Maresova P. Use of social media platforms among adults in the United States—behavior on social media. Societies. 2020;10(1):27. doi: 10.3390/soc10010027

[81] Segado Fernández S, Jiménez Gómez B, Jiménez Hidalgo P, Lozano-Estevan MDC, Herrera Peco I. Disinformation about diet and nutrition on social networks: A review of the literature. Nutr Hosp. 2025. doi: 10.20960/nh.05533 40008658

[82] Denniss E, Lindberg R, Marchese LE, McNaughton SA. #Fail: The quality and accuracy of nutrition-related information by influential Australian Instagram accounts. Int J Behav Nutr Phys Act. 2024;21(1):16. doi: 10.1186/s12966-024-01565-y 38355567 PMC10865719

[83] Cheshire A, Berry M, Fixsen A. What are the key features of orthorexia nervosa and influences on its development? A qualitative investigation. Appetite. 2020;155:104798. doi: 10.1016/j.appet.2020.104798 32717291

[84] Lofft Z. When social media met nutrition: How influencers spread misinformation, and why we believe them. Health Sci Inq. 2020;11(1):56–61. doi: 10.29173/hsi319

[85] Vasconcelos C, Costa RLD, Dias ÁL, Pereira L, Santos JP. Online influencers: Healthy food or fake news. IJIMA. 2021;15(2):149. doi: 10.1504/ijima.2021.114334

[86] Culbert KM, Racine SE, Klump KL. Research Review: What we have learned about the causes of eating disorders - A synthesis of sociocultural, psychological, and biological research. J Child Psychol Psychiatry. 2015;56(11):1141–64. doi: 10.1111/jcpp.12441 26095891

[87] Media effects of experimental presentation of the ideal physique on eating disorder symptoms: A meta-analysis of laboratory studies - ScienceDirect. Accessed April 17, 2025. https://www.sciencedirect.com/science/article/abs/pii/S0272735812001638 10.1016/j.cpr.2012.10.011 23232051

[88] Boone L, Soenens B, Luyten P. When or why does perfectionism translate into eating disorder pathology? A longitudinal examination of the moderating and mediating role of body dissatisfaction. J Abnorm Psychol. 2014;123(2):412–8. doi: 10.1037/a0036254 24886015

[89] Sedera D, Lokuge S. Flaws in flawlessness: Perfectionism as a new technology driven mental disorder. 2020. doi: 10.48550/ARXIV.2010.12032

[90] Frost RO, Heimberg RG, Holt CS, Mattia JI, Neubauer AL. A comparison of two measures of perfectionism. Personal Individ Differ. 1993;14(1):119–26. doi: 10.1016/0191-8869(93)90181-2

[91] Huynh PA, Miles S, Nedeljkovic M. Perfectionism as a moderator of the relationship between orthorexia nervosa and obsessive-compulsive symptoms. Eat Weight Disord. 2024;29(1):6. doi: 10.1007/s40519-023-01629-1 38198059 PMC10781834

[92] Barlow IU, Lee E, Saling L. Orthorexia nervosa versus healthy orthorexia: Anxiety, perfectionism, and mindfulness as risk and preventative factors of distress. Eur Eat Disord Rev. 2024;32(1):130–47. doi: 10.1002/erv.3032 37670425

[93] Miley M, Egan H, Wallis D, Mantzios M. Orthorexia nervosa, mindful eating, and perfectionism: An exploratory investigation. Eat Weight Disord. 2022;27(7):2869–78. doi: 10.1007/s40519-022-01440-4 35829900 PMC9556414

[94] Williamson DA, White MA, York-Crowe E, Stewart TM. Cognitive-behavioral theories of eating disorders. Behav Modif. 2004;28(6):711–38. doi: 10.1177/0145445503259853 15383683

[95] Stott N, Fox JRE, Williams MO. Attentional bias in eating disorders: A meta-review. Int J Eat Disord. 2021;54(8):1377–99. doi: 10.1002/eat.23560 34081355

[96] Rowlands K, Wilson E, Simic M, Harrison A, Cardi V. A critical review of studies assessing interpretation bias towards social stimuli in people with eating disorders and the development and pilot testing of novel stimuli for a cognitive bias modification training. Front Psychol. 2020;11:538527. doi: 10.3389/fpsyg.2020.538527 33101116 PMC7556207

[97] Iceta S, Rodrigue C, Legendre M, Daoust J, Flaudias V, Michaud A, et al. Cognitive function in binge eating disorder and food addiction: A systematic review and three-level meta-analysis. Prog Neuropsychopharmacol Biol Psychiatry. 2021;111:110400. doi: 10.1016/j.pnpbp.2021.110400 34256024

[98] Ioannidis K, Taylor C, Holt L, Brown K, Lochner C, Fineberg NA, et al. Problematic usage of the internet and eating disorder and related psychopathology: A multifaceted, systematic review and meta-analysis. Neurosci Biobehav Rev. 2021;125:569–81. doi: 10.1016/j.neubiorev.2021.03.005 33713700

[99] Keegan E, Tchanturia K, Wade TD. Central coherence and set-shifting between nonunderweight eating disorders and anorexia nervosa: A systematic review and meta-analysis. Int J Eat Disord. 2021;54(3):229–43. doi: 10.1002/eat.23430 33305366

[100] Matheson E, Wade TD, Yiend J. Utilising cognitive bias modification to remedy appearance and self-worth biases in eating disorder psychopathology: A systematic review. J Behav Ther Exp Psychiatry. 2019;65:101482. doi: 10.1016/j.jbtep.2019.101482 31170622

[101] Tenconi E, Meregalli V, Buffa A, Collantoni E, Cavallaro R, Meneguzzo P, et al. Belief inflexibility and cognitive biases in anorexia nervosa-the role of the bias against disconfirmatory evidence and its clinical and neuropsychological correlates. J Clin Med. 2023;12(5):1746. doi: 10.3390/jcm12051746 36902532 PMC10003469

[102] Balzan RP, Gilder M, Nancarrow T, Mavrangelos T, Wade TD. Hasty decision-making in individuals at higher risk of developing an eating disorder. J Behav Ther Exp Psychiatry. 2022;75:101717. doi: 10.1016/j.jbtep.2021.101717 34929485

[103] Dent E, Martin AK. Negative comments and social media: How cognitive biases relate to body image concerns. Body Image. 2023;45:54–64. doi: 10.1016/j.bodyim.2023.01.008 36812802

