# Supplementary material for: The association between orthorexia nervosa and social media use: A systematic review and meta-analysis
Source: PLOS Ment Health. 2026 Oct 21;3(10):e0000758. doi: 10.1371/journal.pmen.0000758 (PMC13686286; doi:10.1371/journal.pmen.0000758)
Supplement: Supplementary file 1 [file pmen.0000758.s001.docx]

Date: 5/ jan/ 2025

Table A: Pubmed

| Search | Query | Item Found |
| --- | --- | --- |
| #1 | ("Orthorexia Nervosa"[Mesh]) OR (Nervosa, Orthorexia) OR (Obsessive Healthy Eating) OR (Healthy Eating, Obsessive) OR (Orthorexia) | 757 |
| #2 | ("Social Media"[Mesh]) OR (Media, Social) OR (Social Medium) OR (Web 2.0) OR (Web 2.0s) OR (Twitter Messaging) OR (Messaging, Twitter) OR (Mobile Social Networks) OR (Mobile Social Network) OR (Social Network, Mobile) OR (Mobile Social Media) OR (Mobile Social Medias) OR (Social Media, Mobile) OR (Social Tagging) OR (Social Taggings) OR (Tagging, Social) OR (Folksonomy) OR (Folksonomies) OR (Social Media Messaging) OR (Media Messaging, Social) OR (Messaging, Social Media) OR (Social Media Messagings) | 86,575 |
| #3 | #1 AND #2 | 60 |

Table B: Embase

| Search | Query | Item Found |
| --- | --- | --- |
| #1 | 'orthorexia nervosa'/exp OR 'orthorexia nervosa' OR 'nervosa, orthorexia' OR 'obsessive healthy eating' OR 'healthy eating, obsessive' OR 'orthorexia' | 737 |
| #2 | 'social media'/exp OR 'social media' OR 'media, social' OR 'social medium' OR 'web 2.0' OR 'web 2.0s' OR 'twitter messaging' OR 'messaging, twitter' OR 'mobile social networks' OR 'mobile social network' OR 'social network, mobile' OR 'mobile social media' OR 'mobile social medias' OR 'social media, mobile' OR 'social tagging' OR 'social taggings' OR 'tagging, social' OR 'folksonomy' OR 'folksonomies' OR 'social media messaging' OR 'media messaging, social' OR 'messaging, social media' OR 'social media messagings' | 87,534 |
| #3 | #1 AND #2 | 69 |

Table C: Web of Science

| Search | Query | Item Found |
| --- | --- | --- |
| #1 | TS=("Orthorexia Nervosa" OR "Nervosa, Orthorexia" OR "Obsessive Healthy Eating" OR "Healthy Eating, Obsessive" OR "Orthorexia") | 658 |
| #2 | TS=("Social Media" OR "Media, Social" OR "Social Medium" OR "Web 2.0" OR "Twitter Messaging" OR "Mobile Social Networks" OR "Mobile Social Media" OR "Social Tagging" OR "Folksonomy" OR "Social Media Messaging") | 143,329 |
| #3 | #1 AND #2 | 48 |

Table D: Scopus

| Search | Query | Item Found |
| --- | --- | --- |
| #1 | TITLE-ABS-KEY("Orthorexia Nervosa" OR "Nervosa, Orthorexia" OR "Obsessive Healthy Eating" OR "Healthy Eating, Obsessive" OR "Orthorexia") | 2,433 |
| #2 | TITLE-ABS-KEY("Social Media" OR "Media, Social" OR "Social Medium" OR "Web 2.0" OR "Twitter Messaging" OR "Mobile Social Networks" OR "Mobile Social Media" OR "Social Tagging" OR "Folksonomy" OR "Social Media Messaging") | 817,853 |
| #3 | #1 AND #2  ( ALL ( "Orthorexia Nervosa" OR "Nervosa, Orthorexia" OR "Obsessive Healthy Eating" OR "Healthy Eating, Obsessive" OR "Orthorexia" ) ) AND ( ALL ( "Social Media" OR "Media, Social" OR "Social Medium" OR "Web 2.0" OR "Twitter Messaging" OR "Mobile Social Networks" OR "Mobile Social Media" OR "Social Tagging" OR "Folksonomy" OR "Social Media Messaging" ) ) | 681 |

Table E: PsycInfo

| Search | Query | Item Found |
| --- | --- | --- |
| #1 | (DE "Orthorexia Nervosa" OR "Orthorexia Nervosa" OR "Nervosa, Orthorexia" OR "Obsessive Healthy Eating" OR "Healthy Eating, Obsessive" OR "Orthorexia") | 414 |
| #2 | (DE "Social Media" OR "Social Media" OR "Media, Social" OR "Social Medium" OR "Web 2.0" OR "Twitter Messaging" OR "Mobile Social Networks" OR "Mobile Social Media" OR "Social Tagging" OR "Folksonomy" OR "Social Media Messaging") | 40 299 |
| #3 | #1 AND #2 | 22 |
