# Supplementary material for: The association between orthorexia nervosa and social media use: A systematic review and meta-analysis
Source: PLOS Ment Health. 2026 Oct 21;3(10):e0000758. doi: 10.1371/journal.pmen.0000758 (PMC13686286; doi:10.1371/journal.pmen.0000758)
Supplement: Supplementary file 2 [file pmen.0000758.s003.docx]

**NOS Risk of Bias Assessment**

| **Author (Year)** | **Title** | **Representativeness of sample** | **Sample size** | **Non-Responders** | **Risk tool** | **Comparability** | **Outcome assessment** | **Statistical Test** | **Total Score (Risk of bias)** |
| --- | --- | --- | --- | --- | --- | --- | --- | --- | --- |
| Dobos (2024) | Mindful eating and orthorexia nervosa: How do they interact? | - | - | - | ** | ** | * | * | 6 (Moderate) |
| Awad (2024) | Association of Social Media Use Disorder and Orthorexia Nervosa Among Lebanese University Students: The Indirect Effect of Loneliness and Factor Structure of the Social Media Use Disorder Short Form and the Jong-Gierveld Loneliness Scales | - | * | - | ** | ** | * | * | 7 (Low) |
| Staskiewicz-Bartecka (2024) | The Impact of Social Media and Socio-Cultural Attitudes toward Body Image on the Risk of Orthorexia among Female Football Players of Different Nationalities | * | - | - | ** | - | * | * | 5 (Moderate) |
| Kalender (2024) | A Study on Health Nutrition Obsession, Social-Physique Anxiety, and Social Media Addiction of Young Individuals | * | * | - | ** | - | * | - | 5 (Moderate) |
| Piko (2024) | Orthorexic Tendency in Light of Eating Disorder Attitudes, Social Media Addiction and Regular Sporting Among Young Hungarian Women | - | * | - | ** | ** | * | * | 7 (Low) |
| Blackburn (2024) | #ForYou? the impact of pro-ana TikTok content on body image dissatisfaction and internalisation of societal beauty standards | - | * | - | ** | ** | * | * | 7 (Low) |
| Unal (2024) | The Mediating Role of Attitudes Towards Eating in the Relationship Between Compulsive Instagram Use and Orthorexia in Adults. | - | - | - | ** | ** | * | * | 6 (Moderate) |
| Bayram (2024) | Exploring the interplay between social media addiction, mindful eating, intuitive eating, orthorexia nervosa, and mental health in young adults | - | * | - | ** | ** | * | * | 7 (Low) |
| Sener (2023) | Investigation of the Relationship Between Social Media Addiction and Orthorexia Nervosa in Adult Individuals Who Applied to Obesity Polyclinic | - | - | - | ** | - | * | * | 4 (Moderate) |
| Silva (2023) | The body dissatisfaction role in the adoption of compulsive healthy eating behaviors | - | - | - | ** | ** | * | * | 6 (Moderate) |
| Asil (2023) | The effect of social media use on orthorexia nervosa: a sample from Turkey | - | * | - | ** | * | * | * | 6 (Moderate) |
| Karniej (2023) | Orthorexia nervosa in gay men-the result of a spanish-polish eating disorders study | - | - | - | ** | ** | * | * | 6 (Moderate) |
| Levin (2023) | Examining orthorexia nervosa: Using latent profile analysis to explore potential diagnostic classification and subtypes in a non-clinical sample | - | - | - | ** | ** | * | * | 6 (Moderate) |
| Scheiber (2023) | Socio-cultural power of social media on orthorexia nervosa: An empirical investigation on the mediating role of thin-ideal and muscular internalization, appearance comparison, and body dissatisfaction | - | - | - | ** | ** | * | * | 6 (Moderate) |
| Tarsitano (2022) | Symptoms of Orthorexia Nervosa are associated with time spent on social media: a web-based survey in an Italian population sample | - | * | - | ** | ** | * | * | 7 (Low) |
| Yurtdas-Depboylu (2022) | The association between social media addiction and orthorexia nervosa, eating attitudes, and body image among adolescents | * | - | - | ** | ** | * | * | 7 (Low) |
| Gobin (2021) | The Effects of the COVID-19 Pandemic Lockdown on Eating, Body Image, and Social Media Habits Among Women With and Without Symptoms of Orthorexia Nervosa | - | - | - | ** | - | * | * | 4 (Moderate) |
| Yilmazel (2021) | Orthorexia tendency and social media addiction among candidate doctors and nurses | - | * | - | ** | ** | * | * | 7 (Low) |
| Turner (2017) | Instagram use is linked to increased symptoms of orthorexia nervosa | - | - | - | ** | ** | * | * | 6 (Moderate) |
| Daniel  (2025) | Relationships Between Orthorexia, Exercise Dependency, Body Image, and Decision-Making in University Undergraduates | - | - | - | * | - | * | * | 3 (High) |
| Zagreb  (2025) | Biological, Behavioural, Psychological and Social Predictors of Orthorexia Nervosa Among Young Women in Croatia | * | - | - | ** | ** | * | * | 7 (Low) |
| Kus  (2025) | From likes to obsessions: The mediating role of obsessive compulsive symptoms in the relationship between social media addiction and orthorexia nervosa | * | * | - | ** | - | * | * | 6 (Moderate) |
| Biyikli  (2024) | Evaluation of Orthorexia Nervosa Tendency and Social Media Addiction in University Students | * | * | - | ** | - | * | * | 6 (Moderate) |
| Christodoulou  (2024) | Exploring the Link between Mindful Eating, Instagram Engagement, and Eating Disorders: A Focus on Orthorexia Nervosa | * | - | - | ** | * | * | * | 6 (Moderate) |
| Hamurcu  (2023) | The correlation between orthorexia nervosa and social media use in nursing students | - | * | - | ** | - | * | * | 5 (Moderate) |
| Ulutaş (2025) | The type of social media is a greater influential factor for orthorexia nervosa than the duration: a cross-sectional study. | - | * | - | ** | ** | * | * | 7 (Low) |
| Kovan (2025) | Social media use, eating attitudes, orthorexia nervosa and well-being: testing a moderated mediation model. | - | * | - | ** | - | * | * | 5 (Moderate) |
| Toğuç (2025) | Psychosocial effects of social media and nutrition information: an analysis of orthorexia nervosa and weight-related self-stigma in youth. | - | * | - | ** | ** | * | * | 7 (Low) |
| Ruiz-Lazaro (2025) | The Role of Social Media Addiction, Depression, Rumination and Disordered Eating Behaviours in Orthorexia Nervosa Symptoms. | * | - | - | ** | - | * | * | 5 (Moderate) |
| Thayuman (2025) | Nonattachment to self mediates the relationship between Instagram engagement and orthorexia nervosa amongst Australian university students. | - | - | - | ** | * | * | * | 5 (Moderate) |
| Boutouis & Grant 2025 | Problematic usage of the internet is associated with orthorexia nervosa tendency in adults | - | - | - | ** | ** | * | * | 6 (Moderate) |
| Calpbinici 2025 | Effect of social media use on the orthorexia nervosa tendency among pregnant women | - | * | - | ** | ** | * | * | 7 (Low) |
| Adiningsih et al. 2024 | Effect of social media use on the orthorexia nervosa tendency among pregnant women | - | - | - | ** | - | * | * | 4 (Moderate) |
